# The full transcription map of mouse papillomavirus type 1 (MmuPV1) in mouse wart tissues

**DOI:** 10.1371/journal.ppat.1006715

**Published:** 2017-11-27

**Authors:** Xiang-Yang Xue, Vladimir Majerciak, Aayushi Uberoi, Bong-Hyun Kim, Deanna Gotte, Xiongfong Chen, Maggie Cam, Paul F. Lambert, Zhi-Ming Zheng

**Affiliations:** 1 Tumor Virus RNA Biology Section, RNA Biology Laboratory, Center for Cancer Research, NCI/NIH, Frederick, Maryland, United States of America; 2 Department of Microbiology and Immunology, Wenzhou Medical University, Zhejiang, China; 3 McArdle Laboratory for Cancer Research, University of Wisconsin School of Medicine and Public Health, Madison, Wisconsin, United States of America; 4 Collaborative Bioinformatics Resource, Center for Cancer Research, NCI/NIH, Bethesda, Maryland, United States of America; 5 Frederick National Laboratory for Cancer Research, Leidos Biomedical Research, Frederick, Maryland, United States of America; Stony Brook University, UNITED STATES

## Abstract

Mouse papillomavirus type 1 (MmuPV1) provides, for the first time, the opportunity to study infection and pathogenesis of papillomaviruses in the context of laboratory mice. In this report, we define the transcriptome of MmuPV1 genome present in papillomas arising in experimentally infected mice using a combination of RNA-seq, PacBio Iso-seq, 5’ RACE, 3’ RACE, primer-walking RT-PCR, RNase protection, Northern blot and *in situ* hybridization analyses. We demonstrate that the MmuPV1 genome is transcribed unidirectionally from five major promoters (P) or transcription start sites (TSS) and polyadenylates its transcripts at two major polyadenylation (pA) sites. We designate the P_7503_, P_360_ and P_859_ as “early” promoters because they give rise to transcripts mostly utilizing the polyadenylation signal at nt 3844 and therefore can only encode early genes, and P_7107_ and P_533_ as “late” promoters because they give rise to transcripts utilizing polyadenylation signals at either nt 3844 or nt 7047, the latter being able to encode late, capsid proteins. MmuPV1 genome contains five splice donor sites and three acceptor sites that produce thirty-six RNA isoforms deduced to express seven predicted early gene products (E6, E7, E1, E1^M1, E1^M2, E2 and E8^E2) and three predicted late gene products (E1^E4, L2 and L1). The majority of the viral early transcripts are spliced once from nt 757 to 3139, while viral late transcripts, which are predicted to encode L1, are spliced twice, first from nt 7243 to either nt 3139 (P_7107_) or nt 757 to 3139 (P_533_) and second from nt 3431 to nt 5372. Thirteen of these viral transcripts were detectable by Northern blot analysis, with the P_533_-derived late E1^E4 transcripts being the most abundant. The late transcripts could be detected in highly differentiated keratinocytes of MmuPV1-infected tissues as early as ten days after MmuPV1 inoculation and correlated with detection of L1 protein and viral DNA amplification. In mature warts, detection of L1 was also found in more poorly differentiated cells, as previously reported. Subclinical infections were also observed. The comprehensive transcription map of MmuPV1 generated in this study provides further evidence that MmuPV1 is similar to high-risk cutaneous beta human papillomaviruses. The knowledge revealed will facilitate the use of MmuPV1 as an animal virus model for understanding of human papillomavirus gene expression, pathogenesis and immunology.

## Author summary

Papillomavirus (PV) infections lead to development of both benign warts and cancers. Because PVs are epitheliotropic and species specific, it has been extremely challenging to study PV infection in the context of a naturally occurring infection in a tractable laboratory animal. The recent discovery of the papillomavirus, MmuPV1, that infects laboratory mice, provides an important new animal model system for understanding the pathogenesis of papillomavirus-associated diseases. By using state of the art RNA-seq to provide deep sequencing analysis of what regions of the viral genome are transcribed and PacBio Iso-seq that produces longer reads to define the complete sequences of individual transcripts in combination with several conventional technologies to confirm transcription starts sites, splice sites, and polyadenylation sites, we provide the first detailed description of the MmuPV1 transcript map using RNA from MmuPV1-induced mouse warts. This study reveals the presence of mRNA transcripts capable of coding for ten protein products in the MmuPV1 genome and leads to correctly re-assigning the E1^E4, L2 and L1 coding regions. We were able to detect individual transcripts from the infected wart tissues by RT-PCR, Northern blot and RNA ISH, to define the temporal onset of productive viral infection and to ectopically express a predicted viral protein for functional studies. The constructed MmuPV1 transcript map provides a foundation to advance our understanding of papillomavirus biology and pathogenesis.

## Introduction

Human papillomaviruses are a group of small, non-enveloped, epitheliotropic DNA tumor viruses whose infection can result in benign lesions (called warts or papillomas) and in some cases cause malignancies. Certain genotypes of HPVs, such as HPV-16, HPV-18, and HPV-31, that infect mucosal epithelia, have been recognized as causative agents of anogenital cancers that include cervical and anal cancers, as well as a growing subset of head and neck cancers, particularly those arising in the oropharynx [1,2]. Papillomaviruses are species-specific. Although papillomavirus infection models in large animal species such as rabbits, dogs and cows have been used to study the molecular biology and pathogenesis of papillomavirus infections, a laboratory mouse model would greatly facilitate the study of papillomavirus-associated warts and cancers. The recent identification of the murine papillomavirus (MmuPV1) that can infect laboratory strains of mice now provides us with such a tractable laboratory animal-based infection model system [3–6]. The MmuPV1 circular, double stranded DNA genome is 7510-bp in length and encodes at least seven translational open reading frames (ORFs), designated E1, E2, E4, E6, E7, L1 and L2 based upon their conserved position within the viral genome and length comparable to ORFs of other papillomaviruses [7,8]. To date, a transcription map of MmuPV1 has not been described. Such a map would greatly facilitate understanding the MmuPV1genome structure and viral gene expression capacities and thereby information on nature of viral factors that contribute to papillomavirus infection and associated pathogenesis.

In this report, we describe a comprehensive map of MmuPV1 transcripts based upon a multi-pronged analysis of viral mRNAs isolated from tumor tissues derived from MmuPV1 infected mice. The viral transcription start sites (TSS) were mapped by 5’ rapid amplification of cDNA ends (5’-RACE) [9,10] in combination with PacBio Iso-seq and confirmed by TA cloning and Sanger sequencing. The polyadenylation cleavage sites of viral early and late transcripts were mapped by 3’-RACE [9,10]. Viral genome expression and RNA splicing was profiled by RNA-seq and primer-walking RT-PCR [9–11]. Accordingly, we assigned the coding regions of multiple potential viral gene products, E1^E4, E1^M1, E1^M2, E8^E2, L2 and L1 based upon the deduced structure of mRNA transcripts, and performed in *situ* hybridization studies in which we could detect a subset of these viral early and late transcripts in MmuPV1 infection-derived wart tissues by RNA-ISH in correlation with the presence of viral proteins and viral genomic DNA.

## Results

### MmuPV1 infection and genome expression profile in wart tissues

**Fig 1:**
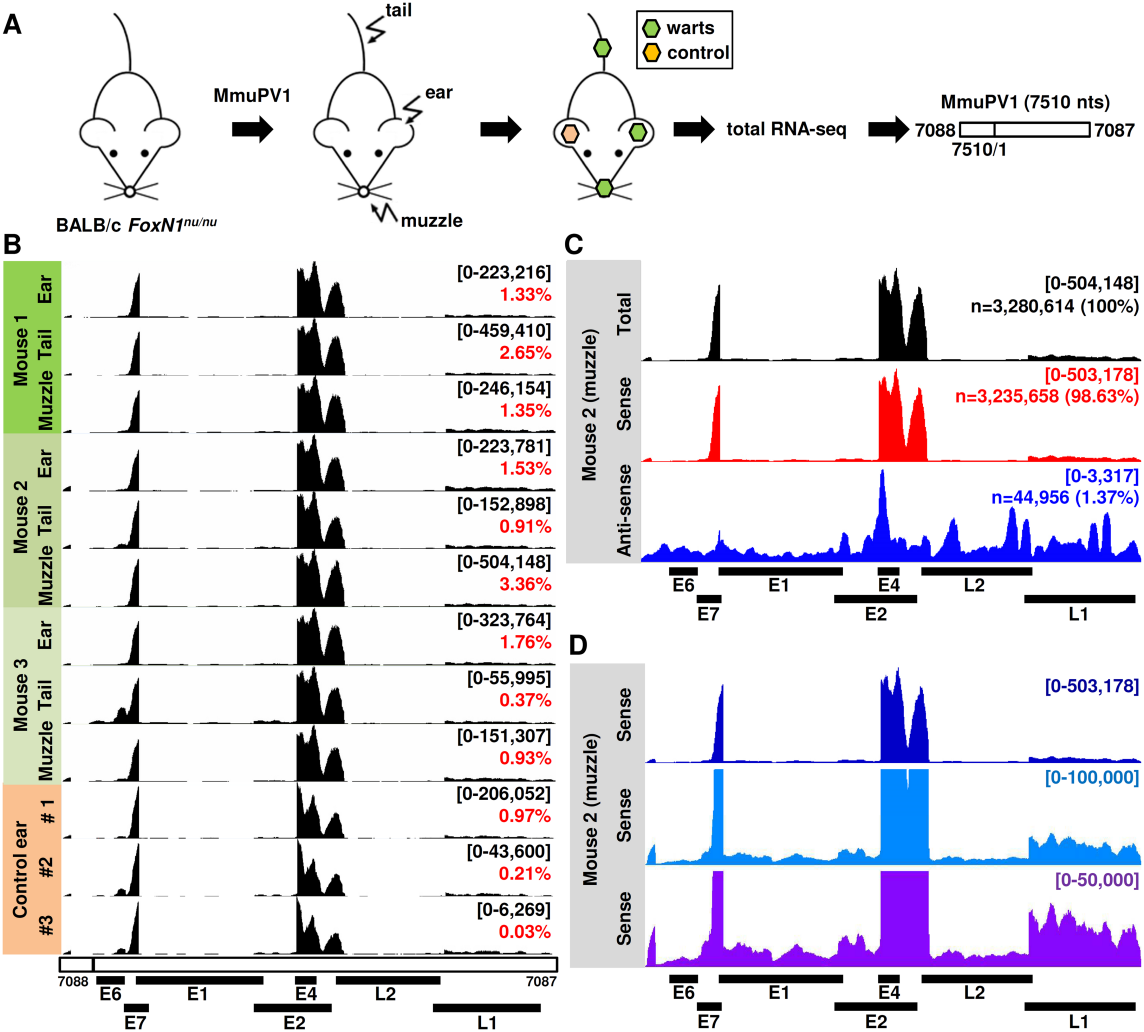
RNA-seq analyses of MmuPV1 gene expression from wart tissues. (A) Schematic depicting experimental MmuPV1 infection of immunodeficient athymic BALB/c *FoxN1*^*nu/nu*^ mice. The wart tissues were dissected for total RNA extraction followed by strand-specific RNA-seq analysis. The obtained sequence libraries were mapped to MmuPV1 reference genome (NC_014326) starting at nt 7088. (B-D) Coverage and distribution of MmuPV1-specific reads along with the viral genome as visualized by IGV software. The scales in upper right corner shows the coverage depth of viral reads set to autoscale unless specified otherwise. Numbers in red are % viral RNA reads from total number of RNA reads. Below are positions of the annotated MmuPV1 open reading frames (ORFs) [4]. (B) Distribution of viral RNA reads from wart tissues on three distinct anatomical sites (ear, tail, and muzzle) of 3 separate animals. Control ear total RNA was extracted from experimentally uninfected ear from the same MmuPV1 wart-bearing animals. (C) Distribution of virus-specific RNA reads based on viral genome DNA strand-specificity from the mouse 2 muzzle tissue which contains the highest number of viral reads. (D) The depth of coverage of viral sense-strand reads in various parts of viral genome at three different scales of coverage.

Athymic *FoxN1*^*nu/nu*^ mice were infected with MmuPV1 as follows: inner (right) ear, muzzle, and three spots on the tail following scarification (Fig 1A). Resulting warts were harvested six months following infection and total RNA was isolated. Papillomatosis was confirmed by histopathological analysis (S1A Fig). Papillomas exhibited fibrillary projections accompanied with hyperkeratosis and were exophytic in morphology. Koilocytes, considered as hallmarks of papillomavirus infection, were seen throughout the papillomas. Productive phase of viral life cycle was confirmed by L1 positivity by immunofluorescence and detection of amplified viral DNA by fluorescence *in situ* hybridization. L1 and amplified viral DNA were detected throughout the papilloma including the terminally differentiating epithelia (S1A Fig).

To elucidate the presence and relative abundance of RNA transcripts arising from the MmuPV1 genome from these wart tissues (S1A Fig), ribosomal RNA-depleted total RNA isolated from each wart tissue at three anatomical sites of three infected animals was analyzed by RNA-seq. In addition, the uninfected ear from the same animal was used as an uninfected control. Approximately 100 million paired-end reads with high quality were obtained from each tissue sample (Table 1). By mapping the RNA-seq raw reads from each lesion to the newly arranged linear MmuPV1 genome starting from nt 7088 and ending at nt 7087 using RNA sequence aligner TopHat [12], we obtained ~0.4–3.3 million viral reads for each wart sample (GEO Accession No. GSE104118), varying among lesions and animals, which accounts for ~0.4%-3.4% of total RNA reads obtained from each sample and with the muzzle tissues from animal #2 containing the most RNA reads (Table 1). Surprisingly, we also obtained many MmuPV1 reads from the uninfected ear tissues (control ears) in all three wart-bearing animals (Table 1), with the animal #1 uninfected ear (left ear) displaying the viral reads similar to that of the infected right ear while appearing normal by visual scoring. MmuPV1 L1 protein and DNA were detected in this tissue confirming the presence of subclinical infections in these control ear tissues (S1B Fig). By uploading these uniquely mapped viral RNA reads obtained from individual samples to the Integrative Genomics Viewer (IGV) program to visualize reads coverage profile along with the MmuPV1 genome, we found three major coverage peaks, one in the E7 region, one in the E4 region and one between E4 and L2, make the last two peaks as a V shape (Fig 1B) among all wart-tissues and sub-clinically infected ear tissues obtained from the MmuPV1 wart-bearing animals. The V shape was attributed to (1) fewer uniquely mapped viral RNA reads, but more host-viral chimeric reads being excluded from the mapping in this region, and (2) RNA splicing by using a 5’ splice site at nt 3431 (see more detailed description later in this report). We also saw a small drop of viral RNA reads within the E4 ORF in particular in the control animal ears and this might be a result from increased E1^E4 splicing in proportion in the control animal ears when compared with the tumor ear RNA. By analyzing the orientation of the uniquely mapped viral RNA reads from the muzzle tissues obtained from mouse #2 which appeared the highest viral reads coverage, we conclude that the vast majority of the viral reads were of the sense strand (98.6%). Antisense-specific reads were of low abundance (~1.4% of all viral reads), although a few peaks at various points along with the viral genome were evident (Fig 1C). We view these antisense reads being background noise from cDNA library construction, sequencing errors or mapping artifacts. A step-wise zoom-in view further showed that the viral sense transcripts derived from the LCR (long control region), E6, E1, E2, L2 and L1 regions were less abundant than the reads from the E7 and E4 regions, with the L1 reads a little more than the others (Fig 1D).

**Table 1:** Number of MmuPV1 reads in twelve RNA-seq sequence libraries.

Sample	Total reads	Viral reads	% viral reads
**Mouse 1-ear**	98,110,500	1,306,707	1.33
**Mouse 1-tail**	104,483,954	2,773,796	2.65
**Mouse 1-muzzle**	118,337,440	1,602,197	1.35
**Mouse 2-ear**	92,238,944	1,411,045	1.53
**Mouse 2-tail**	103,848,432	949,720	0.91
**Mouse 2-muzzle**	97,560,544	3,280,614	3.36
**Mouse 3-ear**	115,737,714	2,036,580	1.76
**Mouse 3-tail**	108,930,490	400,544	0.37
**Mouse 3-muzzle**	98,428,320	918,794	0.93
**Mouse 1-control ear**	100,128,144	971,642	0.97
**Mouse 2-control ear**	98,049,954	209,678	0.21
**Mouse 3-control ear**	102,481,576	29,509	0.03

### Mapping of MmuPV1 transcription start sites (TSS) for early and late MmuPV1 transcripts

**Fig 2:**
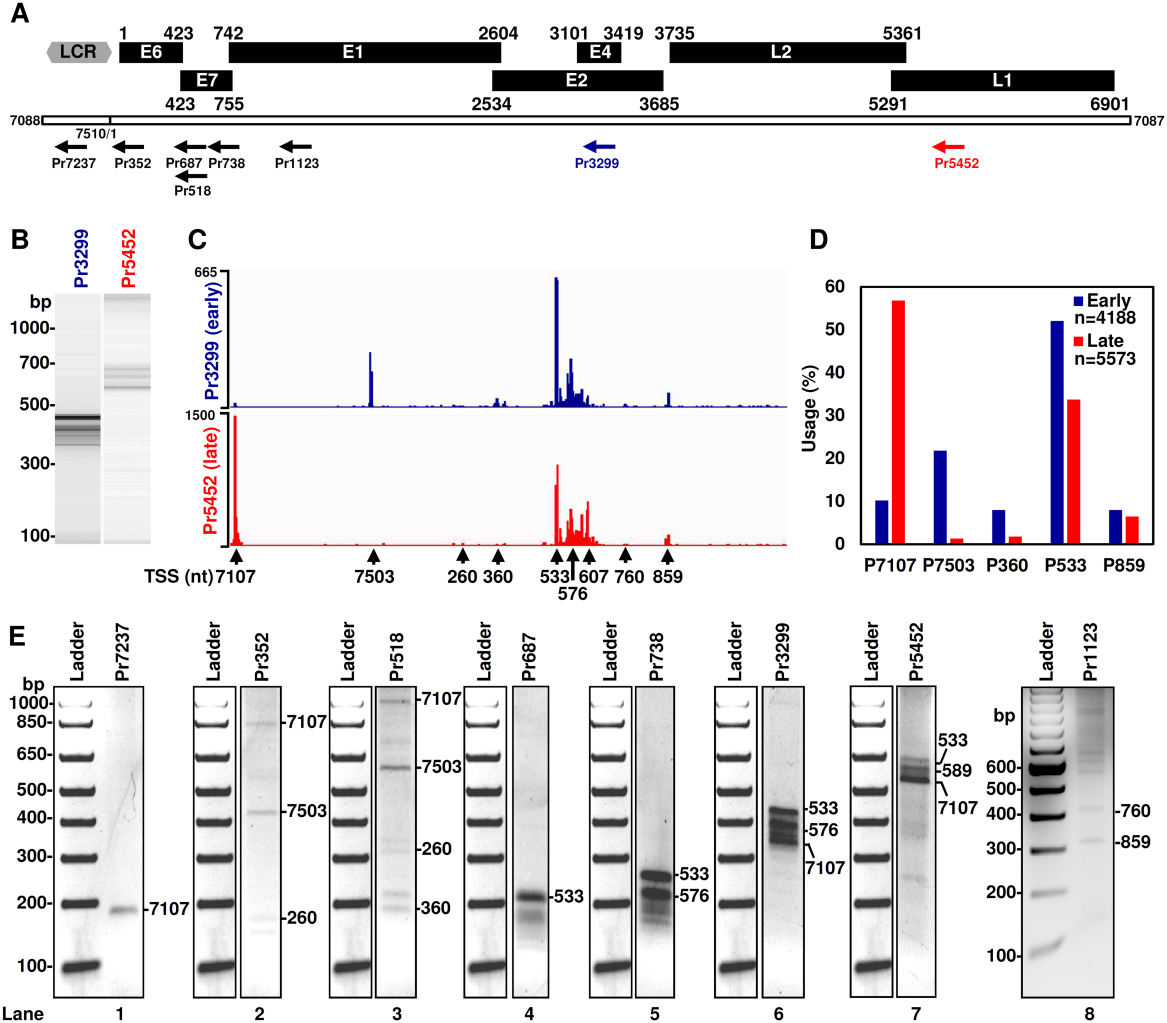
TSS mapping of MmuPV1 transcripts by 5’RACE in combination with PacBio Iso-seq and TA cloning-Sanger sequencing. (A) Schematic diagrams of MmuPV1 ORFs initially deduced from the MmuPV1 genome [4]. The numbers above each ORF are the first nt of the start codon and the last nt of the termination codon (except E4) in the MmuPV1 genome illustrated by a bracket line below the ORFs as a linear form, with the head-to-tail junction with starting position at nt 7088. Notes: the initial assigned E4 ORF does not have a start codon [4] and the nt 3101 above E4 is not the first nt of the start codon for E4, nor a splice acceptor site. LCR, long control region. Primers (arrows) used for 5′ RACE are shown below are named by the positions of their 5′ ends in the virus genome. (B) Size distribution 5’RACE products obtained from mouse wart tissues-derived total RNA by a primer from the early (Pr3299) region and a primer from the late (Pr5452) regions of MmuPV1 genome as shown by Agilent Bioanalyzer. The 5’RACE products were converted to sequence libraries and subjected to single-molecule PacBio Iso-seq sequencing. (C) Profiles of the viral RNA transcription start sites mapped by PacBio Iso-seq. The obtained Iso-seq transcript reads were trimmed of the adaptors and mapped to the reference MmuPV1 genome. The numbers on the left are autoscale showing the reads coverage depth. Below are the major viral TSS identified by traditional 5’ RACE analysis. (D) Frequency (%) of individual viral TSS usage in MmuPV1 infection of the mouse 2 muzzle. The relative frequency (%) was calculated by numbers of the full-length transcript reads with the same 5’ end +/- 10 nt divided by total Iso-seq transcripts mapped to the six major viral TSS. (E) The 5′ RACE products of MmuPV1 transcripts obtained with different MmuPV1-specific primers on total RNA isolated from MmuPV1-infected wart tissues used in RNA-seq analysis. The individual RACE products were gel purified, cloned, and sequenced. The mapped TSS for MmuPV1 transcripts were obtained from the 5’ RACE products originated from S2 Fig and S2 Table.

Considering majority of eukaryotic RNA has a 5’-end cap structure, the current RNA-seq protocol lacks a de-capping step and fragments RNA into 200–350 nt pieces for adaptor ligation and amplification. These methods create an unfavorable bias for adaptor ligation to the RNA 5’ end and deletion of the RNA 5’ end reads smaller than 200 nts, making the RNA-seq unattainable for mapping the RNA start site [13]. To map the transcription start sites (TSS) of MmuPV1 transcripts, 5′ RACE analyses were performed on total RNA isolated from MmuPV1-infected ear lesions using virus-specific antisense primer Pr7237, Pr352, Pr518, Pr687, Pr738, Pr1123, Pr3299, or Pr5452 (Fig 2A, S2 Fig and S1 Table). The 5′ RACE products were visualized either by a DNA Bioanalyzer (Fig 2B) or by agarose gel electrophoresis (Fig 2E and S2 Fig). The 5’ RACE products derived from Pr3299 and Pr5452 were also subjected to long read single-molecular, real-time sequencing using PacBio (Pacific Biosciences) Iso-seq technology (Fig 2C and 2D, S3A Fig), for detection of existing full-length transcripts and overcoming the RNA-seq unfavorable bias on the fragmented RNA 5’ end. In addition, all 5’ RACE products were gel-purified, cloned and sequenced (Fig 2E, S2 and S3B Figs). From PacBio Iso-eq sequencing, approximate five thousands of the full-length viral transcripts were detected from each primer-derived 5’ RACE products (Table 2). By uploading these Iso-seq-identified individual viral transcripts to the IGV program to visualize their coverage profile along with MmuPV1 genome, we found that the Pr3299-derived 5’ RACE products were mainly transcribed from two TSS, one at nt 533 (~50%) and the other at nt 7503 (~20%) in the viral genome, although other scattered TSS positions were also identified immediately downstream of the nt 533 position (Fig 2C and 2D, Table 2). In contrast, the Pr5452-derived 5’ RACE products were predominantly transcribed from the nt 7107 position (>55%) and secondarily from the nt 533 position (>30%) on the viral genome (Fig 2C and 2D, Table 2). Similar to the Pr3299-derived products, the Pr5452-derived 5’ RACE products displayed scattered TSS positions (mostly from the nt 576 to nt 607) immediately downstream of the nt 533 position (Fig 2C). Both 5’ RACE products gave the same TSS products initiating at nt 859 (Fig 2C and 2D), while the TSS at the nt 360 was most associated with Pr3299-derived products (Fig 2C and 2D, S3A Fig and Table 2).

**Table 2:** Number of PacBio Iso-seq reads assigned to individual viral promoters.

TSS	Pr3299 (early)		Pr5452 (late)	
	Counts	%	Counts	%
**P**_**7107**_	427	10.19	3165	56.80
**P**_**7503**_	912	21.76	74	1.33
**P**_**360**_	335	7.99	99	1.77
**P**_**533**_	2181	52.07	1878	33.70
**P**_**859**_	333	7.99	357	6.41
**Total**	4188	100.00	5573	100.00

Each of the preferred TSS was named as a promoter (P)

The TSS of viral transcripts was also assessed by TA cloning and Sanger sequencing of individual primer-derived 5’ RACE products. As shown in Fig 2E, S2 Fig and S2 Table, one 5’ RACE product obtained from the Pr7237 (lane 1) was mapped primarily to the nt 7107 (6/9 colonies). Three RACE products were detected from the Pr352 (lane 2) and they were defined to have TSS at nt 260 (7/14 colonies), nt 7503 (6/10 colonies) and nt 7107 (direct sequencing of PCR products). Pr518 exhibited a similar 5’ RACE product profile, 166-bp larger than that of Pr352. Direct sequencing of four Pr518 5’ RACE products (lane 3) identified TSS at nt 360, 260, 7503 and 7107. The Pr687 and Pr738 also showed a similar 5’ RACE product profile, with the two Pr738-derived 5’ RACE products being 51-nts larger than that of Pr692 (compare lane 4 to lane 5). Direct sequencing showed the main product from Pr687 had a TSS at nt 533 (lane 4). Cloning and sequencing of the Pr738-derived products 1 or 2 indicated that product 1 had a TSS at nt 576/579/589 (7/20 colonies) and product 2 had a TSS at nt 533 (6/21 colonies) (lane 5). The Pr3299 generated two major spliced 5’ RACE products containing splice junctions at nts 757/3139 or nts 7243/3139 (lane 6), with TSS for product 1 mainly placed at nt 7107 (12/18 colonies) and for product 2 at nts 533 or 576 (6/17 colonies). Pr5452 (lane 7) had a similar 5’ RACE profile to that of Pr3299, but gave three faster migrating faint bands. TA cloning and sequencing of the Pr5452 5’ RACE products revealed that its product 1 had a TSS mainly at nt 7107 (3/8 colonies) and product 2 had a TSS between nt 533–589 (6/8 colonies). Both were double spliced products either from nt 7243 to 3139 or from nt 757 to 3139 and then from nt 3431 to 5372. We did not clone and sequence the faster migrating faint bands which are most likely the products of Pr7107 (216 bp), Pr360 (477 bp) and Pr533 (304 bp) being spliced from nt 7243 or nt 757 to 5372. Using the Pr1123 for 5’ RACE (lane 8), cloning and sequencing revealed two smaller products being, respectively, transcribed from the nt 859 (7/12 colonies) and nt 760 (3/5 colonies).

Together, these 5’ RACE experiments identified nt 7107, 7503, 360, 533 and 859 as preferred TSS for MmuPV1 gene expression. The mapped TSS all started at a purine A or G, which is in agreement with conserved TSS having a purine in eukaryotes [14]. The TSS at nt 7503 drives MmuPV1 E6 transcription, the TSS at nt 360 drives MmuPV1 E7 transcription and the TSS at nt 859 drives E2 expression. The TSS at nts 7107 and 533 drive MmuPV1 late transcription. Hereafter, each of the preferred TSS is named as a promoter: P_7107_, P_7503_, P_360_, P_533_ or P_859_. Analyses of the region 5′ to each mapped TSS show that the P_7503_ has a TATA box (a eukaryotic core promoter motif) 25-bp upstream of its TSS, but other two promoters for viral early gene expression do not (S3B Fig). Two viral late promoters either bear a TATA-like box for P_7107_ or a TATA box 110-bp upstream of the promoter P_533_ (S3B Fig). These features of viral promoters perhaps account for the observed heterogeneity of their transcription initiation as seen in the expression of HPV-18 [10] and other eukaryotic genes [15].

The P_7107_ late promoter identified above by 5’ RACE was confirmed by RNase protection assays (RPA) performed on total RNA isolated from MmuPV1-infected lesions using a ^32^P-labeled antisense RNA probe from nt 6846 to 7237 that covers the mapped TSS around nt 7107. The RPA products were analyzed by electrophoresis in a denaturing 8% PAGE gel, along with the DNA sequencing ladders generated from MmuPV1 genome by a ^32^P-labeled antisense primer Pr7237. As shown in S4 Fig, the protected RPA products from the 6846–7237 probe showed two major bands, one (arrow) of 131 nts in length corresponds to the TSS at nt 7107. The other product of 217 nts (arrowhead) corresponds to the L1 mRNA cleaved at nt 7063 for the late polyadenylation (see below).

### Mapping of MmuPV1 polyadenylation cleavage sites for early and late transcripts

**Fig 3:**
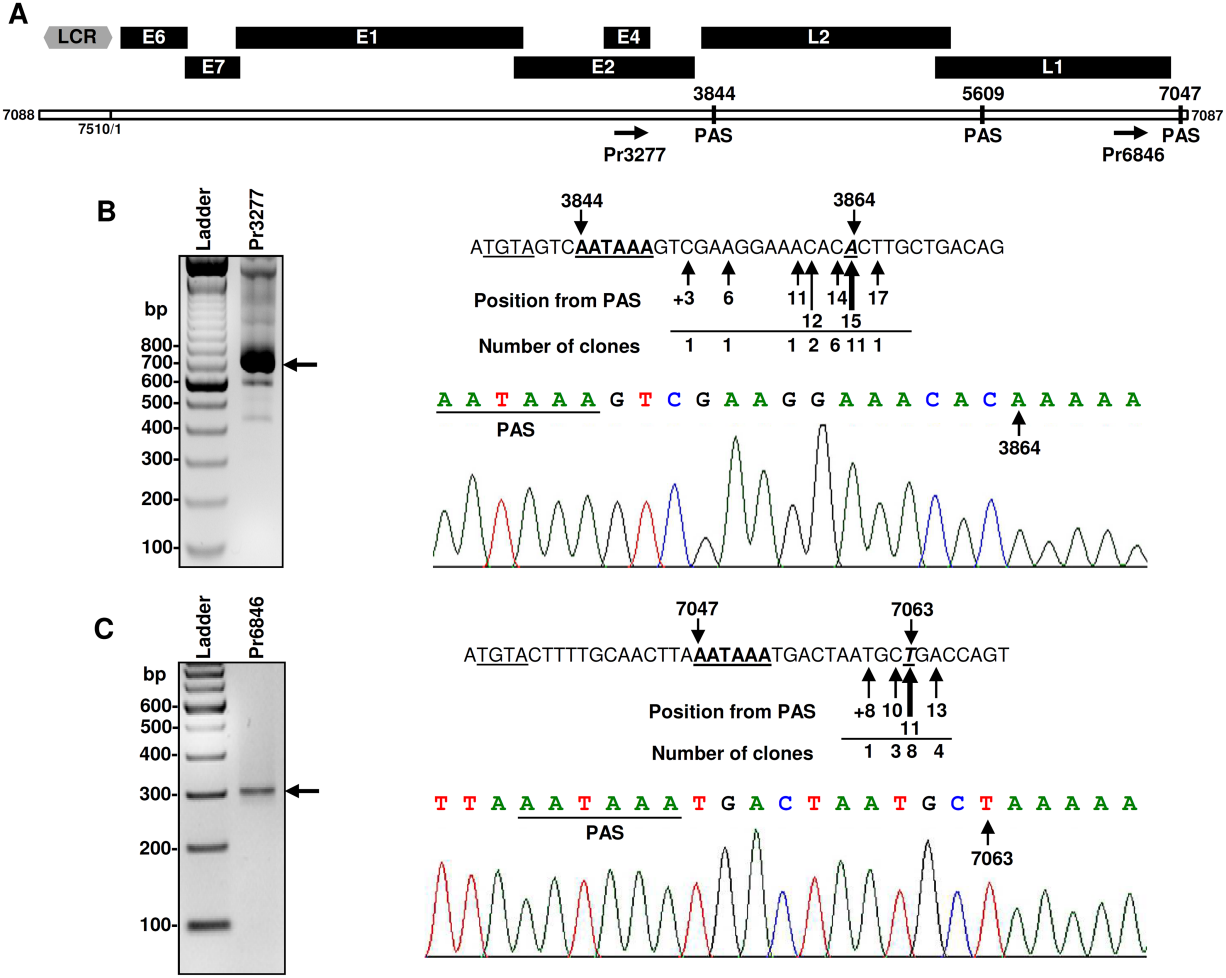
Mapping of polyadenylation cleavage sites for MmuPV1 transcripts. (A) The diagram depicting MmuPV1 genome with the position of primers used in 3’RACE analysis and three predicted canonical polyadenylation signals (PAS). The associated number represents the nt position in the MmuPV1 genome. (B-C) Mapping of MmuPV1 polyadenylation cleavage sites by 3′RACE was conducted on total RNA isolated from MmuPV1-infected wart tissues using MmuPV1-specific primer Pr3277 for viral early (B) or Pr6846 for viral late transcripts (C). The obtained RACE products (arrows) were gel purified, cloned, and sequenced. Sequence readings on the right show frequency and positon of all mapped cleavage sites for early (B) or late (C) viral pA sites. Canonical PAS sequences are bolded and underlined and the putative UGUA motifs (TGTA in cDNA) are underlined. Below is chromatograph of representative sequence showing the position of PAS (underlined) and cleavage site (arrow).

Genome analyses of MmuPV1 suggest a putative early poly(A) signal (PAS) AAUAAA downstream of the viral E2 ORF at nt 3844 presumably for early polyadenylation and two putative late PAS, at nt 5609 and 7047, responsible presumably for polyadenylation of viral L2 and L1 transcripts (Fig 3A). To determine further the early polyadenylation cleavage sites (CS), total RNA isolated from MmuPV1-infected lesions was analyzed by 3′ RACE using a MmuPV1-specific sense primer Pr3277 located within the E4 ORF. Following gel purification, cloning, and sequencing of a 3′ RACE product of ~750 -bp (Fig 3B), we found that 11/23 sequenced bacterial colonies exhibited a product with a 3′-end at nt 3864, 15 nt downstream of the putative nt 3844 PAS (Fig 3B). Additional 3′ RACE with a MmuPV1-specific sense primer Pr116 and Pr522, located within the E6 and E7 ORF, respectively, also determined the early cleavage site around nt 3864, confirming that MmuPV1 early transcripts are cleaved at nt 3864 for RNA polyadenylation using the nt 3844 PAS AAUAAA although its 3′ downstream sequence has no U/GU motifs, the highly conserved recognition sites for CSF (cleavage stimulation factor) binding in context of the RNA polyadenylation [16,17].

To detect the late CS, we carried out a 3′ RACE with MmuPV1-specific sense primers Pr6846 or Pr5433 located in the L1 ORF. Following gel purification and cloning of the Pr6846-derived 3′ RACE products of ~310 bp, we sequenced 16 bacterial colonies and found the usage of multiple cleavage sites for polyadenylation of MmuPV1 late transcripts. Eight clones exhibited a product with a 3′end at nt 7063, 11 nt downstream of the putative nt 7047 PAS AAUAAA motif (Fig 3C). 3′ RACE with an additional MmuPV1-specific sense primer Pr5433 also determined the late CS primarily mapping to nt 7063 (S5 Fig). Analysis of the region downstream of this cleavage site shows three overlapping U/GU motifs from nts 7109 to 7152. RPA analysis of the total cell RNA isolated from MmuPV1-infected lesions using the ^32^P-labeled antisense RNA probe from nt 6846 to 7237 further confirmed the presence of the late CS around the nt 7063 (S4 Fig).

In addition, we identified infrequent usage of the nt 5609 PAS AAUAAA for RNA polyadenylation at nt 5627 from Pr5433-derived 3’ RACE products (S5 Fig). This PAS could be useful for the expression of L2, but would lead to produce a truncated L1 protein. Because of its low frequency of usage, we consider that it represents a cryptic polyadenylation site of unknown function.

### Mapping MmuPV1 exon-exon splice junctions

**Fig 4:**
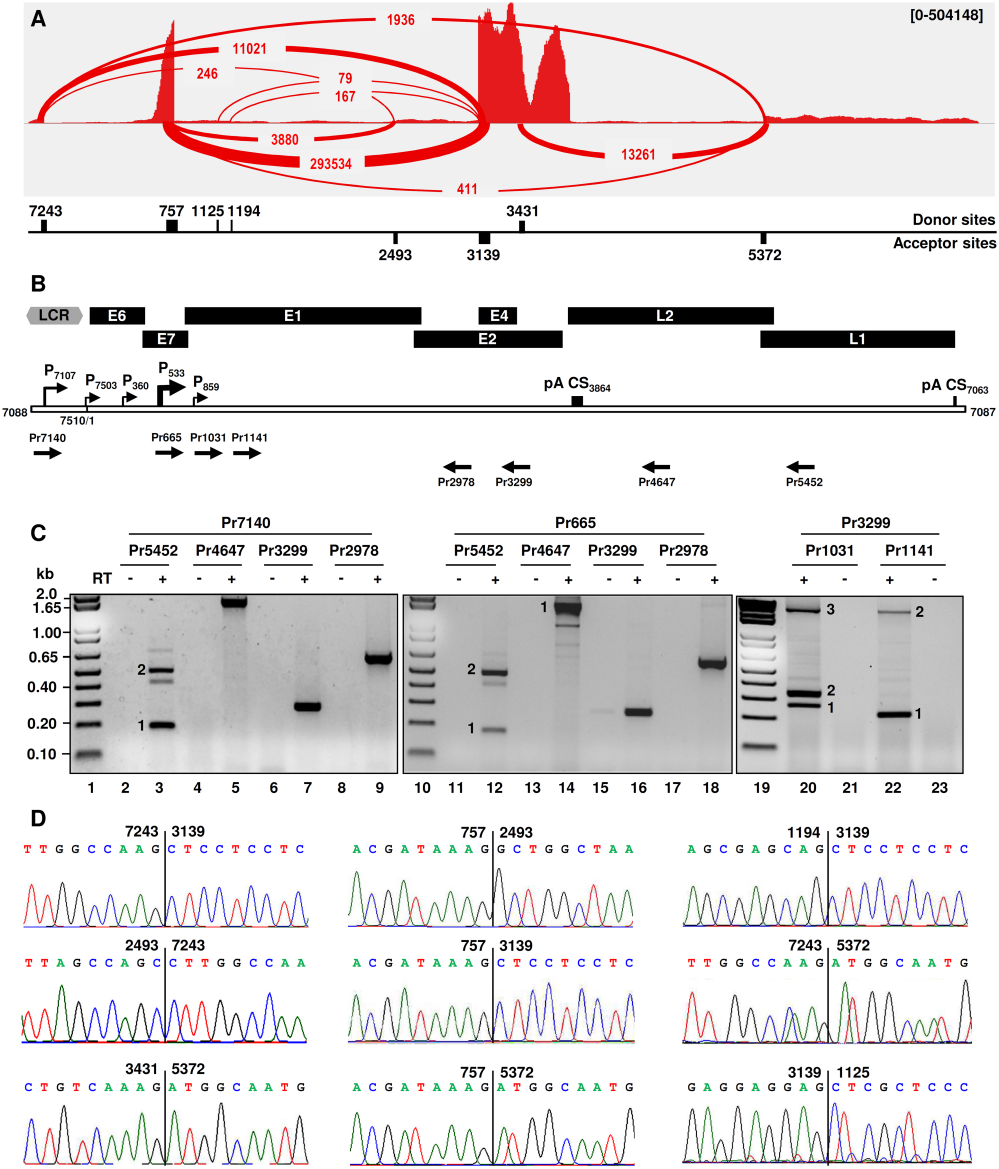
Identification and validation of MmuPV1 splice junctions. (A) Identification of MmuPV1 splice junctions by RNA-seq along with the MmuPV1 genome. The MmuPV1-specific junction reads were identified by STAR (Spliced Transcripts Alignment to a Reference) and GSNAP (Genomic Short-read Nucleotide Alignment Program) aligners and further refined by criteria described in Material and Methods. Sashimi plot was used to visualize the positon and frequency of splice junctions along the viral genome. Below are the nucleotide positions of all identified 5’ (donor) and 3’ (acceptor) splice sites utilized by viral early or late transcripts. (B) Diagram of original ORFs and the mapped promoters and polyadenylation cleavage sites (CS) in the MmuPV1 genome and the positions of primers used for primer-walking RT-PCR analysis. (C-D) Validation of MmuPV1 splice junctions by primer walking RT-PCR. The primer walking RT-PCRs in the presence (+) or absence (-) of RT were performed on total RNA isolated from MmuPV1-infected warts (C). All obtained RT-PCR products shown in (C) were gel-purified, cloned and sequenced. The chromatographs show the representative product sequences with nucleotide positions at the identified splice junction (D).

Given the presence of multiple splice sites in both early and late transcripts of papillomaviruses, we used a snout (muzzle) wart sample from the animal #2 that gave 3.28 million viral reads, the highest among all twelve samples, to elucidate all possible usage of viral splice sites in the MmuPV1 genome. STAR aligner program [18] was used to explore the potential exon-exon splicing junctions with a threshold of minimal overhang >30 nts for non-canonical junctions and >10 nts for canonical junctions. As shown in Fig 4A, we identified from this sample 324,535 junction reads (10% of total viral reads) that defined nine splicing junctions, with frequency by numbers of the junction reads in the order of nts 757/3139 >3431/5372 >7243/3139>757/2493 >7243/5372 >757/5372 >7243/2493 >1194/3139 >1125/3139 (S3 Table). The most common splice junction read, at nts 757/3139, accounted for 90% of total junction reads. The least common reads were those for the 1125/3139 splice, accounting for only 0.02% of the total junction reads. The identified splice junctions of 7243/3139, 757/3139, and 3431/5372 (Fig 4A) confirmed the findings from the 5’ RACE analyses using Pr3299 and Pr5452 primers (Fig 2E). IGV Sashimi plot visualized that the obtained viral junction reads were derived from five different 5’ splice sites (donor sites) to three separate 3’ splice sites (acceptor sites) in the MmuPV1 genome (Fig 4A). Analysis of the intron sequences between the exon-exon junctions confirmed that all introns contain a consensus GU dinucleotide in the intron 5′ end and a consensus AG dinucleotide in the intron 3′ end. The similar frequency patterns of the detected splice junctions were observed in all remaining samples, except the splice junction 1125/3139 which was detected only in 4 out of 9 tumor samples (S3 Table). We noticed that the 757/3139 junction reads were proportionally higher in the sub-clinically infected control ears than that seen in the tumor ears (S3 Table).

Primer walking RT-PCR using various combinations of primer pairs (Fig 4B) on total mRNA isolated from MmuPV1-infected lesions was used to further validate the exon-exon junctions identified by RNA-seq and by 5’ RACE for MmuPV1 early and late transcripts. Gel-purification and sequencing of each RT-PCR product confirmed all of the splice junction identified by RNA-seq. Using a forward primer of Pr7140 downstream of the late promoter P_7107_ in combination with a backward primer of Pr5452 (L1), Pr4647 (L2), Pr3299 (E4) or Pr2978 (E2), as shown in Fig 4C, we detected two L1 products (lane 3) and one each for L2, E4 and E2 (lanes 5, 7 and 9). Sequencing of the gel-purified RT-PCR products indicated that two L1 products (185-bp and 478-bp, lane 3) were the singly spliced (at 7243/5372) and doubly spliced (at 7243/3139 and 3431/5372) transcripts, respectively (Fig 4D); both an L2 product (1612-bp, Fig 4C, lane 5) and an E4 product (265-bp, Fig 4C, lane 7) were singly spliced (7243/3139) transcripts (Fig 4D); and an E2 product (590-bp, Fig 4C, lane 9) was a singly spliced (7243/2493) transcript (Fig 4D). Using a forward primer of Pr665 within the E7 ORF in combination with the same sets of backward primers also showed two L1 products (Fig 4C, lane 12) and one each for L2, E4 and E2 (Fig 4C, lanes 14, 16 and 18). L1 product 1 (174-bp, Fig 4C, lane 12) was a singly spliced (757/5372) transcript, whereas the L1 product 2 (467-bp, Fig 4C, lane 12) was a doubly spliced (757/3139 and 3431/5372) transcript (Fig 4D); both L2 (1602-bp) and E4 (254-bp) products (Fig 4C, lanes 14 and 16) were singly spliced (757/3139) transcripts; the E2 product (579-bp, Fig 4C, lane 18) was a singly spliced 757/2493 transcript (Fig 4D).

Using a backward primer Pr3299 in combination of a forward primer Pr1031 or Pr1141, we detected three RT-PCR products in sizes of 257-bp (product 1), 326-bp (product 2) and 2269-bp (product 3) for Pr1031 and two products of 214-bp (product 1) and 2158-bp (product 2) for Pr1141 (Fig 4C, lanes 20 and 22). We found both 257-bp and 326-bp products were the spliced products of 1125/3139 and 1194/3139 and the 214-bp product was also a spliced product of 1194/3139 (Fig 4D). Both the 2269-bp and 2158-bp products were unspliced products from this region presumably for E1 expression.

### Viral intron retention during MmuPV1 genome expression

**Fig 5:**
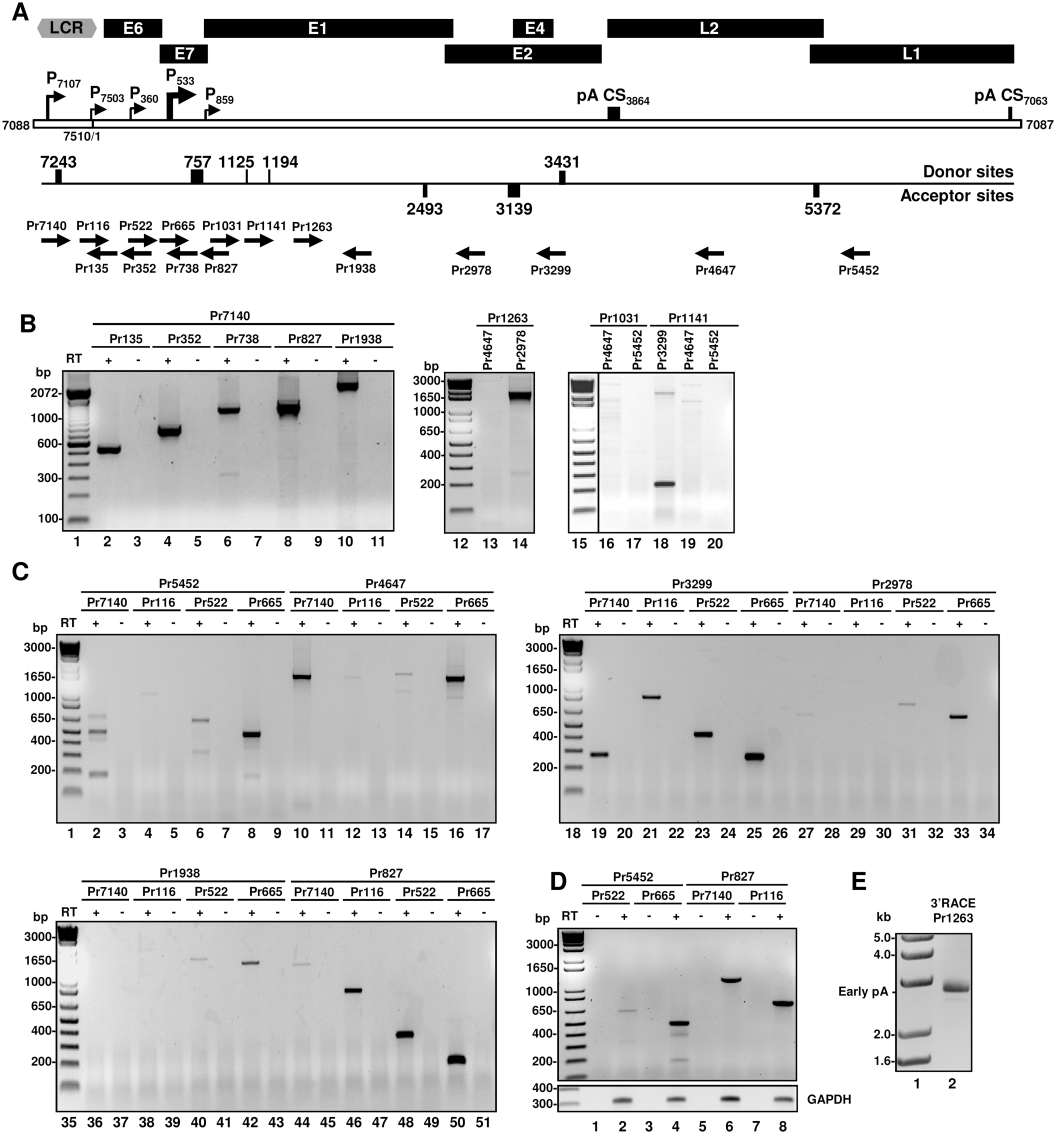
Intron retention and abundance of unspliced viral transcripts. (A) A diagram of MmuPV1 genome (a bracket line in the middle) with original assigned ORFs and a long control region (LCR), along with the identified splice sites, mapped viral promoters (P), polyadenylation CS sites (pA CS) and the position and orientation of primers used in the primer-walking study. Each primer was named by a number representing the position of the primer’s 5’end in the MmuPV1 genome. (B-C) The primer walking RT-PCRs were performed in the presence (+) or absence (-) of RT on total RNA isolated from the muzzle wart tissues of animal #2 and the obtained products were gel-purified and sequenced. PCR results in (B) were from 35 cycles and in (C) from 25 cycles and were all amplified from the same cDNA. (D) RT-PCR detection for intron retention and abundance of unspliced MmuPV1 RNA on total RNA isolated from the ear wart tissues of animal #3. GAPDH RNA served as a sample loading control. PCR results were from 25 cycles. (E) The 3’RACE performed with intron-based E1-specific Pr1263 on cDNA from the muzzle tissues of animal #2. The predicted 3’ RACE product for E1 transcripts is ~2.6 kb when polyadenylated at nt 3864 position.

Intron retention is one of the common alternative splicing events during RNA splicing and is essential for the expression of E1 and L2 in all papillomaviruses. In high-risk HPVs, intron retention is also necessary for viral E6 expression. From RNA-seq analysis, a small fraction of viral reads spanned over the entire MmuPV1 E1 and L2 ORF regions (Figs 1D and 4A). These were further confirmed by primer-walking RT-PCR with a series of combined primer pairs (Fig 5A) from total RNA extracted from the MmuPV1-infected muzzle wart tissues of animal #2. As shown in Fig 5B, the primer-walking RT-PCR using a forward primer Pr7140 in combination with a reverse primer Pr135, Pr352, Pr738, Pr827 or Pr1938 for detection of the P_7107_-derived late transcripts, which are predominantly spliced from the nt 7243 5’ss to nt 3139 3’ss, led to the amplification of transcripts with an intron retained from nt 7244 to 3138 (lanes 2, 4, 6, 8 and 10). Using a forward primer Pr1263 in combination with a backward primer Pr4647 for L2 or Pr2978 for E2 (lanes 13–14), we obtained a 1715-bp product (lane 14) from the Pr2978, but none from the Pr4647 (lane 13), indicating retention of the intron within the E1 and E2 ORFs, but not together with retention of another intron in the L2 region. Using a forward primer of Pr1141 in combination with a backward primer of Pr3299, we detected a 214-bp product spliced from nt 1194 to 3139 (lane 18). The other primer pairs used in the same detection did not give any obvious RT-PCR products (lanes 16, 17, 19 and 20).

Next, we compared the primer-walking RT-PCR using fewer amplification cycles (25 cycles) and using an exonic or intronic backward primer in combination with the same sets of forward primers (Pr7140, Pr116, Pr522 or Pr665) to detect intron-containing viral transcripts derived from individual promoters in the same muzzle wart tissues. As shown in Fig 5C, we detected two major spliced products from the exonic backward primer Pr5452 (L1) plus the forward primer Pr7140, Pr522 or Pr665 (lanes 2, 6 and 8), the larger amplicon arose from double splicing (either 7243/3139 or 757/3139 and then 3431/5372) and the smaller amplicon arose from single splicing (7243/5372 or 757/5372), depending on the promoter usage. In this case, only a very few products could be amplified from the primer pair of Pr5452 plus Pr116 (lane 4). However, the primer pair of Pr5452 plus Pr522 gave much less amount of the spliced L1 products (lane 6) over the primer pair of Pr5452 plus Pr665 (lane 8), but relatively more than the primer pair of Pr5452 plus Pr116 (lane 4), the data suggest that L1 messages are the spliced product primarily transcribed from two promoters P_7107_ and P_533_. But few L1 might be also derived from the promoter P_360_. The identity of a faint band above 650-bp size from the primer pair of Pr5452 and Pr7140 (lane 2) was unknown and might be nonspecific. When an intronic backward primer Pr4647 in the L2 region was used in combination with the same sets of the forward primers, we obtained only one major product predominantly transcribed from two promoters P_7107_ and P_533_ (lanes 10 and 16) and spliced from 7243/3139 (P_7107_) or 757/3139 (P_533_), but very little from the promoter P_7503_ or P_360_ (lanes 12 and 14), suggesting that these two promoter-derived transcripts are used for L2 production. To detect intron retention in the viral early region required for the expression of E1, the primer-walking RT-PCR results from an exonic backward primer Pr3299 (lanes 19–26) were compared with backward primers Pr2978 (lanes 27–34), Pr1938 (lanes 36–43) or Pr827 (lanes 44–51) in combination of the same sets of primers described above for the detection. We found that the Pr3299 amplified one single spliced RT-PCR product of 7243/3139 for P_7107_-derived transcripts (lane 19) or 757/3139 for P_7503_-, P_360_- or P_533_-derived transcripts (lanes 21, 23 and 25). The Pr2978 detected a single spliced, weak product of 7243/2493 for P_7107_-derived transcripts (lane 27) or 757/2493 for P_360_- (lane 31), but a little more of 757/2493 for P_533_-derived transcripts (lane 33) and none from the promoter P_7503_ (lane 29). The Pr1938 detected at low levels an unspliced E1 transcripts derived from the P_360_ promoter (lane 40), but more from the P_533_ promoter (lane 42) and none from promoter P_7107_ (lane 36) or P_7503_ (lane 38). In contrast, the primer Pr827 in combination of each forward primer led to detection of an E1 intron-containing product mostly from three promoters P_7503_, P_360_ and P_533_ (lanes 46, 48 and 50), but little from P_7107_ (lane 44). We also confirmed in an MmPV1-induced ear wart of animal #3 that the L1 messages were the spliced products primarily transcribed from the promoter P_533_, but less from the promoter Pr_360_ (compare lanes 2 and 4 in Fig 5D with lanes 6 and 8 in Fig 5C). Moreover, the intron-containing E1 transcripts were confirmed to be derived from the promoter Pr7107 and Pr7503 (lanes 6 and 8 in Fig 5D and lanes 44 and 46 in Fig 5C). Further studies using an E1-specific primer Pr1263 for 3’ RACE demonstrated that the unspliced E1 transcripts are polyadenylated at nt 3864 position, an early CS site (Fig 5E).

### Construction of a full MmuPV1 transcription map

**Fig 6:**
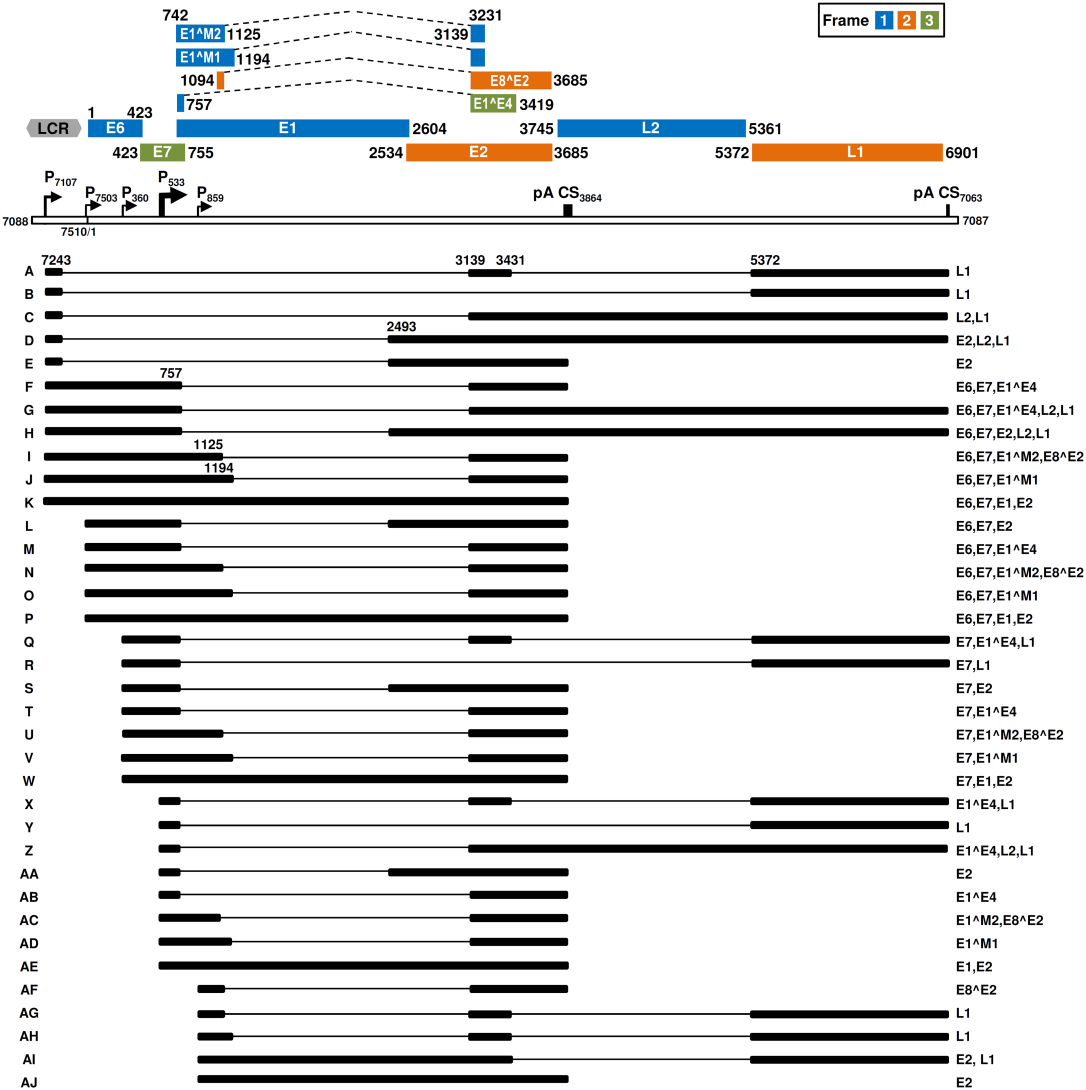
Full transcription map of MmuPV1. The bracket line in the middle of the panel represents a linear form of the virus genome for better presentation of head-to-tail junction, promoters (arrows), and polyadenylation cleavage sites including early pA CS 3864 and late pA CS7063. The open reading frames (ORFs) (color boxes: blue for frame1, yellow for frame 2 and green for frame 3) are diagramed above the bracket line and the numbers above each ORF or on each ORF side are nucleotide positions of the first nucleotide of the start codon and the last nucleotide of the stop codons in the MmuPV1 genome. Dash lines between color boxes indicate the disrupted ORFs by an intron sequence in the virus genome. The split ORFs separated by introns can be formed by splicing of its first exon to a downstream exon either in the same frame (E1^M1, E1^M2 and E8^E2) or in a different frame (E1^E4). LCR indicates a long control region. Below the bracket line are the RNA species derived from alternative promoter usage and alternative RNA splicing. Exons (heavy lines) and introns (thin lines) are illustrated for each species of the RNA, with the mapped splice site positions being numbered by nucleotide positions in the virus genome, and coding potentials on the right.

Based on the mapped TSS, polyadenylation cleavage sites, and RNA splice sites of MmuPV1 early and late transcripts, we constructed a full transcription map from the MmuPV1 genome. As shown in Fig 6. MmuPV1 expresses at least 36 RNA isoforms spanning the entire MmuPV1 genome, with the majority of them being polycistronic transcripts that can potentially translate multiple gene products. MmuPV1 early transcription mainly starts at nt 7503 for E6 polycistronic or nt 360 for E7 polycistronic RNAs. Both are polyadenylated at nt 3864 using a PAS at nt 3844. MmuPV1 late transcription mainly starts either at nt 7107 or nt 533 and polyadenylates either at nt 3864 using the nt 3844 PAS for E1^E4 expression or at nt 7063 using a PAS at nt 7047, the latter encoding L1 and L2 proteins. Most viral early transcripts contain two exons and one major intron spanning the entire E1 ORF and partial E2 ORF. Although the majority of the viral early transcripts have intron 1 spliced out, a small fraction of early transcripts retains this intron (Fig 5B, lane 14, Fig 5C, lanes 40 and 42) and could be further spliced (Fig 4C, lanes 20 and 22; Fig 5B, lane 18 and Fig 5C, lanes 31 and 33). Most viral late transcripts have three exons and two major introns and their 5’ portions overlap with the viral early transcripts. Depending on which late promoter drives transcription, the 5’ end of the first intron can start either at nt 7244 or 758. If starting from nt 758, the first intron of the late transcripts is the same as found in the viral early transcripts. The second intron of the late transcripts is invariably from nt 3432 to nt 5371 and covers the entire L2 ORF and the early PAS. Retention of the intron 2 (Fig 6, products C, D, G, H, Z) and therefore capacity to encode L2 were found only in a small fraction of the late transcripts (Fig 4C, lanes 5 and 14; Fig 5C, lanes 10 and 16). The more abundant viral late transcripts are the ones that encode for E1^E4 and/or L1 (Fig 6, products A, X, AB).

As shown in Fig 6, both MmuPV1 early and late transcripts are alternatively spliced, and the coding capacities of each RNA species may be inferred from the ORF(s) included in the mRNA. We reassigned E4 as E1^E4 with an AUG codon starting from nt 742 position in the viral transcript exon 1 instead of the nt 3101 from a previous publication [4] and thus expression of the E4 ORF requires RNA splicing. We also reassigned the L2 ORF AUG start codon to be at nt 3745 instead of at nt 3735 in the prior publication [4] and the L1 ORF AUG start codon to be at nt 5372 instead of at nt 5291 in the prior publication [4]. To remove the intron 2 during RNA splicing, the viral late transcript exon 2 is spliced right to nt 5372 of the exon 3 and thus the 5291 AUG codon does not exist in the L1 mRNA after RNA splicing. Moreover, we identified the E8^E2 ORF as a spliced ORF (nt 1094-1125/nt 3139–3685) and two small E1 ORF variants, E1^M1 (nt742-1194/nt3139-3231) and E1^M2 (nt742-1125/nt3139-3231) not previously described. A detailed analysis of the upstream sequences of these ORFs indicated that the first AUG codon of each contains a strong Kozak consensus sequence of either ANNaugN or GNNaugG [19,20].

### Expression profiles of the identified viral transcripts in MmuPV1-induced papillomas

**Fig 7:**
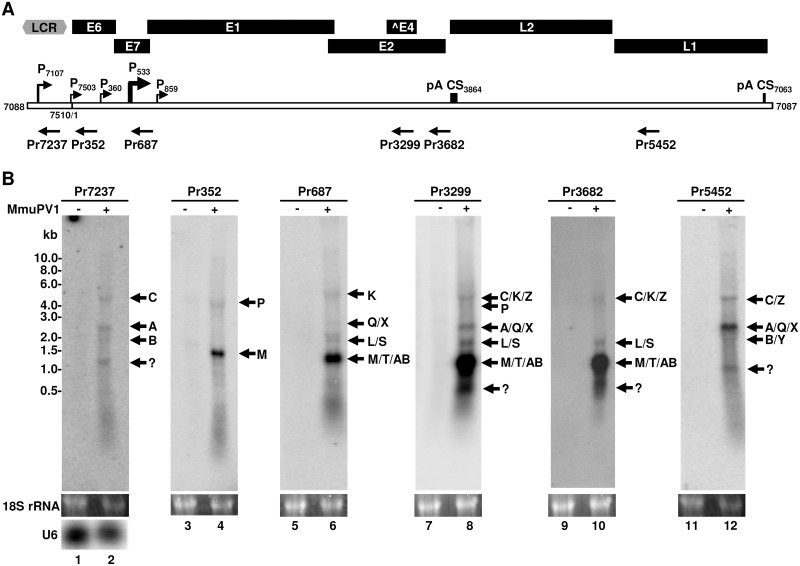
Expression profile of the mapped MmuPV1 transcripts in wart tissues by Northern blot analysis. (A) A diagram showing positions of the identified major TSS (arrows to the right) and polyadenylation CS (pA CS, vertical lines) in the MmupV1 genome (bracket line) and its major ORFs or an incomplete ^E4 ORF (above the bracket line). Below the bracket line are positions of the antisense oligonucleotide probes (arrows to the left) used in Northern blot analysis (B). (B) Profiling the relative expression of the identified individual viral transcripts in MmuPV1-induced wart tissues by Northern blot analysis. The total RNA from mouse ears without MmuPV1 subclinical infection and no detectable MmuPV1 reads by RNA-seq analysis served as a MmuPV1-negative RNA control. Approximate 5 μg of total RNA isolated from uninfected (-) or MmuPV1-infected (+) tissues were separated in a 1% formaldehyde-agarose gel, stained with ethidium bromide for 18S ribosome level as an internal loading control, and transferred onto a nylon membrane. After crosslinking by UV light, the membrane was probed with a ^32^P-labeled antisense oligonucleotide as indicated for MmuPV1 or reprobed with a U6-specific oligo probe.

As shown in Figs 1B and 2E, most RNA reads or transcripts appear to be derived from the promoter P_533_. The relative expression levels of viral transcripts in the infected wart tissues were also measured semi-quantitatively by primer-walking RT-PCR with fewer cycle (25 cycles) amplification (Fig 5C) and the data in this study also suggested that both L1 and L2 are transcribed from either promoter P_7107_ or P_533_ (Fig 5C, lanes 2, 8, 10 and 16), but most viral RNA transcripts are P_533_ transcripts (Fig 5C, compare lane 25 to lanes 23, 21, and 19). To quantify the expression levels of the existing viral transcripts better in the infected tissues, Northern blot analysis using ^32^P-labeled oligo probes, Pr7237, Pr352, Pr687, Pr3299, Pr3682 or Pr5452 (Fig 7A), was further used to evaluate the quantitative levels of the existing viral RNA transcripts from individual promoters. In this study, the total RNA from mouse ears without MmuPV1 subclinical infection served as a MmuPV1-negative RNA control and was pooled RNA from ears of two naïve, freshly arrived female mice in ~4 months of age, with no detectable MmuPV1 reads by RNA-seq analysis. As shown in Fig 7B, we found that the late-transcript-specific probes Pr7237 (lane 2) and Pr5452 (lane 12) in Northern blotting displayed a comparable expression profile of the viral late region as did from the viral early region detected by the early transcript-specific probes Pr687 (lane 6) and Pr3299 (lane 8), with the more hybridization signals seen from the Pr3299 and Pr5452 than the corresponding comparable probes. Size analyses of individual transcripts detected by each probe demonstrated that L1 transcripts of ~2.3-kb in size are predominantly transcribed from the promoter P_7107_ (product A in lanes 2, 8 and 12) or P_533_ (product X in lanes 6, 8 and 12) and are preferentially doubly spliced from nt 7243 to 3139 (P_7107_) or from nt 757 to 3139 (P_533_) and then from nt 3431 to 5372, accompanied by a lower abundance of singly spliced L1 transcripts from nt 7243 (P_7017_, product B in lanes 2 and 12) or nt 757 (P_533_, product Y in lane 12) to 5372. In principle, a minor form of the doubly spliced L1 transcript Q derived from promoter P_360_ also exist in these Northern blot assays (lanes 6, 8 and 12). As expected, these doubly spliced L1 transcripts (A/Q/X) were not detectable by the probe Pr3682 (lane 10) hybridizing to a downstream region of the 3431 5’ donor site. A small fraction of 4.2-kb products (product C in lanes 2, 8, 10 and 12; product Z in lanes 8, 10 and 12) are L2 mRNA arising from nt 7243 splicing to nt 3139 in the case of P_7107_-derived transcripts or from nt 757 splicing to nt 3139 in the case of P_533_-derived transcripts. The ~1-kb L1 products (lanes 2 and 12 in a question mark) appear to be a product of a cryptic PA usage in the L1 ORF (S5 Fig).

As predicted from RNA-seq analysis in Fig 4A, the majority of the P_533_-derived RNA transcripts are spliced from nt 757 to 3139, therefore encoding E1^E4, and utilize the CS at nt 3864 for RNA polyadenylation. As shown in Fig 7B, Northern blotting using ^32^P-labeled oligo probe Pr687 (lane 6) or Pr3299 (lane 8) was able to detect the abundant E1^E4 transcripts (~1.2-kb, products AB) and the minor forms of E6 (~1.4-kb, product M), E7 (~1.1-kb, product T), E2 (~1.8-kb, products L/S), E1 (~4.2-kb, products K or K/P), L1 (~2.3-kb, products Q/X or A/Q/X) and L2 (~4.3-kb, products C and Z). Oligo probe Pr3682 in this assay (Lane 10) displayed the similar detection profile to these two probes except for the doubly spliced L1 which lacks the target sequence for the probe Pr3682 hybridization. Northern blotting using a ^32^P-labeled oligo probe Pr352 exhibited a predominant E6 RNA (~1.4-kb, product M) transcribed from P_7503_ as detected by PacBio Iso-seq (Fig 2C and 2D, Table 2) and spliced from nt 757 to 3139 in addition to the E1 RNA of ~3.9-kb (products P) from this promoter (lane 4). The ~0.8-kb products (lanes 8 and 10 in a question mark) appear to be the products of alternative late promoters around nt 576 to 607 (Fig 2C), which are spliced from nt 757 to nt 3139, but polyadenylated at nt 3864 or are doubly spliced and polyadenylated at a cryptic poly(A) site in the L1 ORF (S5 Fig). Thus, our data from the Northern blotting are consistent with the conclusion from RNA-seq analysis (Fig 4A) and semi-quantitative RT-PCR (Fig 5C) that the majority of viral transcripts are spliced products of 757/3139 and are polyadenylated at nt 3864, using an early PAS at nt 3844 for expression of the early region, and the fewer are polyadenylated at nt 7063, using a late PAS at nt 7047 for expression of the late region.

Subsequently, we also examined viral late transcripts in MuPV1-induced papillomas by RNA-ISH (in situ hybridization). Using antisense probes to the E4 and L1 regions (S6A Fig) and the RNAscope methodology which is highly sensitive in detection of both viral RNA and DNA of MmuPV1, we detected E4 and L1 signals primarily in the highly differentiated granular layers of the infected, hyperproliferative ear skin (S6B Fig). These patterns of E1^E4 and L1 expression were also seen in the tail papillomas (S6C Fig). Although these probes in RNAscope technology detect both viral RNA and DNA, pretreatment of tissue sections with DNase and/or RNase allowed us to distinguish between the DNA-derived and RNA-derived signals using this methodology (S6D and S6E Fig). We found that the detected viral E1^E4 transcripts appeared more cytoplasmic distribution than the L1 transcripts did (S6C Fig), particularly after removal of viral genomic DNA by DNase I treatment of the tissue sections (S6D Fig).

### Spatial localization and expression dynamics of viral gene expression and viral DNA replication in MmuPV1 infected tissues

**Fig 8:**
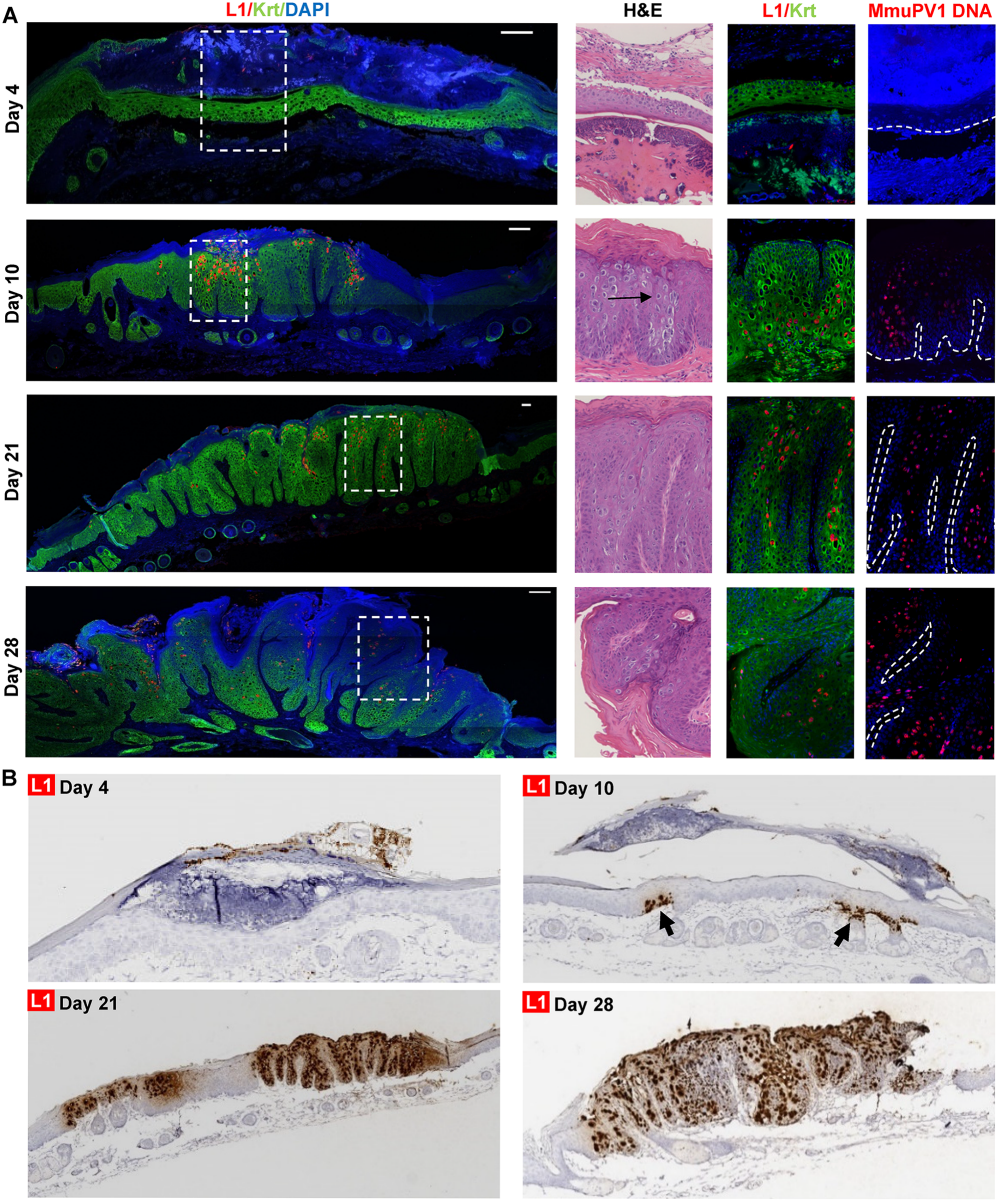
The spatial localization and dynamics of MmuPV1 L1 expression and DNA replication in infected tissues. (A). Time-course analysis of MmuPV1 infected tail site near base of tail of *FoxN1*^*nu/nu*^ mice for L1 protein expression and amplified viral DNA. Each panel consists of a high-resolution wide-field image representing immunofluorescence detection of L1 protein (red) and keratins (Krt10 & Krt14, green) of the infected site accompanied by an inset magnified for H&E staining, L1-Krt immunofluorescence and MmuPV1 DNA FISH. H&E staining and FISH were performed on adjacent sections. The nuclei in IF and FISH were counter-stained with Hoechst for host DNA. Accompanying wide-field images for MmuPV1 DNA FISH staining are provided in S7 Fig. The scale bar for 100 μm is indicated by a white bar. The white dashed lines indicate basement membrane. (B) Time course of MmuPV1 infection and L1 mRNA expression. Tails of nude mice were scarified and infected with 10^8^ VGE of MmuPV1. The infected tail tissues were collected at various days post-infection, fixed, paraffin-embedded, and hybridized with L1-specific antisense probe by RNAscope RNA ISH detection. Note the presence of scar and absence of L1 RNA at day 4.

To understand the expression dynamics of viral L1 and MmuPV1 DNA over time, nude mice were infected (three spots on the tail) with equivalent amounts of MmuPV1 per site (10^8^ VGE) and tissue was harvested by sacrificing animals at different time points until papillomas were overtly seen (28 days post-infection). These tissues collected at each time point were analyzed for appearance of L1 viral protein and amplified viral DNA, hallmarks of the productive phase of the viral life cycle. As shown in Fig 8A and S7 Fig, no obvious L1 expression or viral DNA was detected in any of the infected sites (0/3 infected sites) at 4 days post-infection. Hypertrophic scarring of terminal epithelia with intact basement membrane was observed. At day 10, both L1 and MmuPV1 DNA were detected in one of the infected sites (that closest to base of tail—shown in Fig 8A). L1 expression was found in the suprabasal layers along with terminally differentiating epithelia whereas MmuPV1 DNA appeared to be present only in suprabasal layers. Microscopically, this site had evidence for hyperplasia, koilocytes and some fibrillary projections; however, the overt appearance was that of a raised scar. At day 21, again one site of infection (shown in Fig 8A), that closest to the tail base, showed evidence of productive infection with an abundance of L1-positive and viral DNA-positive cells and more prominent fibrillary projections. The remaining 2 infected sites appeared mostly normal and no L1 or FISH positive nuclei were detected. At 28 days post infection, all three infected sites showed overt appearance of warts with L1 positivity (S8 Fig). While sites were infected at the same time, papillomas grew asynchronously as is evident by variation in size of the papillomas. We consistently observe that papillomas near the base of the tail grow fastest and show the most robust features of a productive viral infection. The pattern of L1 expression and viral DNA amplification at day 28 was similar to that observed at day 21 (Fig 8A). A similar timing and pattern of detection of viral RNA/DNA species was observed using the RNAscope *in situ* hybridization methodology with a probe to the L1 region (Fig 8B).

### Expression of individual MmuPV1 ORFs in HEK293 and HeLa cells

**Fig 9:**
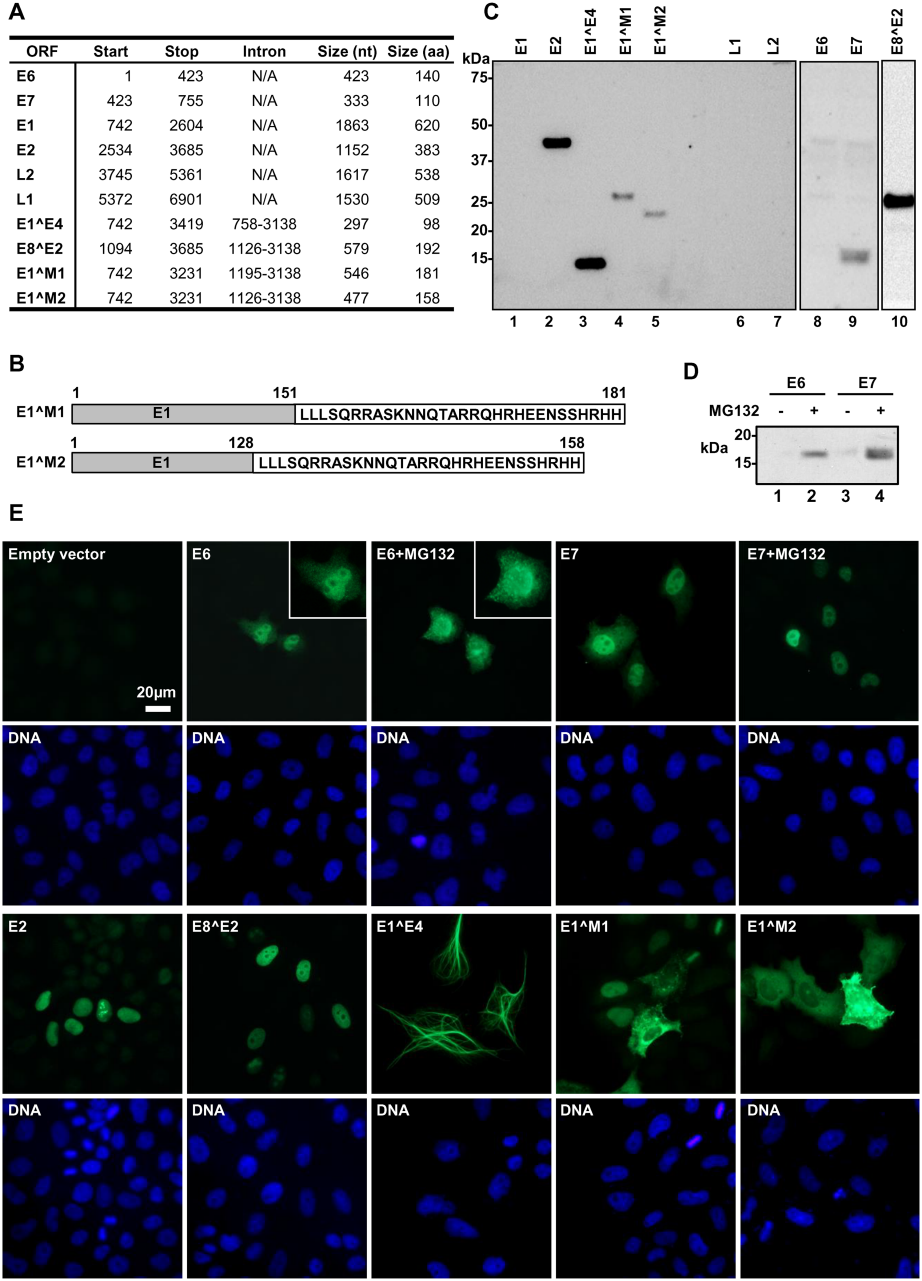
Ectopic expression of the assigned MmuPV1 ORFs in HEK293 cells. (A) MmuPV1 ORFs and their encoded protein size. (B) Protein sequences of E1^M1 and E1^M2. The N-terminal aa sequences of both E1^M1 and E1^M2 (grey box) are the same as the N-terminal E1 protein, but the N-terminal E1 in the E1^M1 is 23 aa residues longer than E1^M2 (grey box). The C-terminal aa residues of both E1^M1 and E1^M2 are the same and encoded by the same exon 2 being off-frame from E1 and thus totally differ from E1. (C) Expression of individual ORFs in HEK293 cells. Individual ORFs assigned were separately inserted into a mammalian expression vector in frame with a C-terminal FLAG-tag. Expression of the corresponding protein in HEK293 cells was examined by Western blotting using an anti-FLAG antibody at 24 h after transfection. (D) Expressed viral E6 and E7 are labile proteins in HEK293 cells. Cell lysates were prepared from HEK293 cells transfected by an E6 or E7 expression vector in the presence or absence of proteasome inhibitor MG132 for 6 h and examined by Western blotting as in (C). (E) FA staining of E6, E7, E2, E8^E2, E1^E4, E1^M1 and E1^M2 in HeLa cells by anti-FLAG M2 monoclonal antibody in combination with Alexa Flour488-labeled anti-mouse secondary antibody. The cell nuclei were counterstained by Hoechst 33342 dye.

Next, we attempted to express each of the predicted MmuPV1 proteins based upon the transcript map in HEK293 and HeLa cells transiently transfected with individual FLAG-tagged viral ORF cDNA expression vectors, including MmuPV1 ORF E6 (GenBank Accession #MF350298), E7 (GenBank Accession #MF350299), E1 (GenBank Accession #MF350300), E2 (GenBank Accession #MF350301), L2 (GenBank Accession #MF350302), L1 (GenBank Accession #MF350303), E1^E4 (GenBank Accession #MF350304), E8^E2 (GenBank Accession #MF350305), E1^M1 (GenBank Accession #MF350306) and E1^M2 (GenBank Accession #MF350307) (Fig 9A and 9B). As shown in Fig 9C, we were able to detect the expression of E2 (lane 2), E1^E4 (lane 3), E1^M1 (lane 4), E1^M2 (lane 5), E7 (lane 9) and E8^E2 (lane 10) in HEK293 cells, but were unable to detect E1 (lane 1), L1 (lane 6), L2 (lane 7) and E6 (lane 8) by FLAG-specific immunoblot analysis. However, both viral E6 and E7 could be better detected when HEK293 cells were treated with proteasome inhibitor MG132 (Fig 9D), indicating they are likely degraded via the proteasome. We also failed to express E1, L1 and L2 in mouse epithelial keratinocytes (S9A Fig) and HEK293TT or HEK293FT cells. By Northern blot analysis of the total RNA extracted from transfected HEK293 cells, we were unable to detect both L1 and L2 RNA, but identified two spliced E1 RNA in smaller sizes and all other expected sizes of viral ORF-derived RNA transcripts (S9B Fig). Using FLAG-specific immunofluorescence (Fig 9E), we detected the expression of E6, E7, E2, and E8^E2 mainly in the nucleus of HeLa cells, E1^E4 as a filamentous protein in the cytoplasm, E1^M1 either in the cytoplasm or nucleus or both, and E1^M2 primarily in the cytoplasm. Distribution of viral E6 and E7 in the cells could be only slightly altered in the presence of MG132 (Fig 9E).

## Discussion

Understanding of the structure and coding capacity of transcripts is critical for disclosing genome function and biology of any organism. In this report, we have utilized two cutting-edge technologies, RNA-seq and PacBio Iso-seq, in combination with various conventional technologies to analyze the structure and expression of MmuPV1 genome in MmuPV1-induced wart tissues with productive MmuPV1 infection. We have constructed the first full transcription map for MmuPV1 and demonstrated that MmuPV1 genome encodes ten ORFs and utilizes five major promoters, two polyadenylation sites and eight splice sites for its expression of thirty-six RNA isoforms during virus infection. Similar to other papillomaviruses [8,10,21–28], this nature of the genome structure with alternative usage of promoters, pA sites and splice sites empowers a highly compact viral genome to express multiple gene products in a temporally and spatially organized manner within its viral life cycle.

The delineated MmuPV1 genome structure and expression of MmuPV1 is more close to that of bovine papillomavirus type 1 (BPV-1), cottontail rabbit papillomavirus (CRPV), cutaneous HPVs and some low-risk mucosotropic HPVs. Like all other papillomaviruses [7,8], the MmuPV1 genome transcribes unidirectionally from one DNA strand, with ~99% of RNA-seq reads mapping to viral sense transcripts. Similar to cutaneous HPVs and low-risk mucosotropic HPVs [25,29,30], MmuPV1 employs two separate early promoters for expression of viral E6 and E7, the promoter P_7503_ for the E6 expression and the promoter P_360_ for the E7 expression. These two promoters could be also responsible for expression of other viral early proteins, including E1, E1^M1, E1^M2, E2 and E8^E2. This strategy for expression of MmuPV1 E6 and E7 is different from high-risk HPVs in that their expression of both viral E6 and E7 is exerted by a single early promoter upstream of the E6 ORF [10,31,32] and the expression of viral E7 requires RNA splicing of an E6 intron in this early transcript [19,33,34]. Also similar to low-risk HPVs [25,29,30] and other animal papillomaviruses [23,24], MmuPV1 has no intron in the E6 ORF region. We found that the promoter P_7503_ is the only early promoter bearing a classical TATA box (a eukaryotic core promoter motif for binding of RNA polymerase II) 25-nts upstream of the promoter TSS. Based on our 5’ RACE and PacBio Iso-seq data, we conclude that the P_7503_ is stronger than the other two early promoters, P_360_ and P_859_. The third early promoter P_859_ is a weak promoter most likely driving the expression of E2 and E8^E2. Because of anticipated RNA-seq bias on eukaryotic RNA 5’ ends [13], fewer RNA reads next to the P_7503_ and P_360_ promoters were noticed (Fig 4A).

MmuPV1 transcribes its late transcripts from two late promoters, P_7107_ in the LCR region downstream of L1 ORF and P_533_ in the E7 ORF region. Utilization of a late promoter in the LCR region for the expression of viral late gene L1 and L2 is a characteristic feature for BPV-1 [22], CRPV [24] and some skin-tropic HPVs such as HPV-1 [35,36] and HPV-5 [29], but not for Mastomys natalensis papillomavirus (MnPV) [23] and other HPVs [10,30–32]. In contrast, high-risk HPVs express their late genes mainly from a late promoter in the E7 ORF [10,31,32,37]. Similar to high-risk HPVs, the late promoter P_533_ in the MmuPV1 genome is most likely responsible for E1^E4 expression, but also for L1 and L2 expression. Although the transcripts derived from promoter P_360_ or P_859_ might have the potential to encode L1, they were scarcely detectable from the infected tissues and could be negligible. We found that the P_7107_ has a TATA-like box 57-nts upstream of its TSS and the P_533_ bears a TATA box at 110-nts upstream of its TSS.

Posttranscriptional RNA processing, including RNA capping, splicing, polyadenylation and export, provides multiple layers of regulation to guide efficient expression of eukaryotic genes [38,39]. By using 3′ RACE, we mapped the cleavage sites of both viral early and late transcripts for RNA polyadenylation and demonstrated that viral early transcripts are polyadenylated primarily at nt 3864 by using a PAS at nt 3844 and viral late transcripts are polyadenylated primarily at nt 7063 by using a PAS at nt 7047. In addition, we have identified a few late transcripts being polyadenylated from nt 5627 by using a PAS at nt 5609 in the L1 ORF. Analyses of the sequences 3’ downstream of each mapped CS site for a highly conserved recognition site U/GU for CSF (cleavage stimulation factor) binding in RNA polyadenylation [16,17] showed three U/GU motifs in this region of the nt 7063, but not so for the nt 3864. Thus, what motif guides the polyadenylation cleavage of the nt 3864 remains unknown. Nevertheless, the mapped polyadenylation site usage for the expression of MmuPV1 early and late transcripts resembles to that of all papillomaviruses [8,10,22–24,29,30,32].

Extensive alternative RNA splicing contributes to the expression of multiple genes by papillomaviruses [8,10,22–24,29,30,32]. Our study revealed this feature in MmuPV1 gene expression by analyzing exon-exon splice-junction reads from RNA-seq and by primer-walking RT-PCR analyses of RNA extracted from MmuPV1-induced warts. We demonstrated that MmuPV1 employs five 5’ splice sites (donor sites) and three 3’ splice sites (acceptor sites) for expression of both viral early and late genes from its five promoters and produces at least thirty-six different RNA isoforms by alternative RNA splicing, of which thirteen were detectable from wart tissues by less sensitive Northern blotting. Thus, the primary MmuPV1 transcripts could have 2 or 3 exons and 1 or 2 introns, with viral E1 and L2 residing in the most commonly excised introns, as seen in other papillomaviruses. As with other papillomaviruses, a small fraction of MmuPV1 RNA transcripts does retain the capacity to express viral E1 and L2; the mechanism by which these RNA transcripts retain the introns for expression of the E1 or L2 should be an attractive area for future investigation.

By construction of this full transcription map, we conclude that MmuPV1 has the potential to express 10 gene products: E6, E7, E1, E1^M1, E1^M2, E1^E4, E2, E8^E2, L2 and L1. Like cutaneous HPVs [40], MmuPV1 does not contain an E5 ORF and therefore no E5 gene product is predicted. The coding regions for the E1^E4, L2 and L1 gene products have been reassigned from what was originally predicted based solely on viral DNA sequence information [4]. E1^M1, E1^M2 and E8^E2 have not been described before for MmuPV1. The E1^M1 and E1^M2 in MmuPV1 might be similar to the E1Ma and E1M in HPV-11 [25]. The MmuPV1 E8^E2 gene product is likely similar to the E8^E2 gene products characterized in BPV-1 [41–44] and HPVs [45,46] and predicted to be encoded by most papillomaviruses [47]. Although the first AUG codon for each of the predicted coding regions has a strong Kozak sequence [20], we were unable to detect the expression of MmuPV1 E1, L2 and L1 proteins from a common eukaryotic expression vector in HEK293, MEK, 293FT or 293TT cells and unable to detect by Northern blotting a full-length RNA expressed from E1, L2 and L1 vector in HEK293 cells. Several possible reasons for this, based upon studies of other papillomaviruses, include rare codon usage [48–50], RNA splicing or instability [8,51,52] and protein stability [53–56]. For example, the E1 ORF in the expression vector contains three splice donor sites (nt 757, nt 1125 and nt 1194 splice donor sites) and one acceptor site (nt 2493 splice acceptor site). The RNA expressed from this vector in HEK293 cells that we could detect by Northern analysis was all spliced forms; no unspliced mRNA capable of expressing full-length E1 could be detected. Among seven viral proteins detected, MmuPV1 E6 and E7 were also found to be increased in their steady state by using a proteasome inhibitor, suggestive that they are normally subjected to proteasomal degradation as seen for HPV-16 [53].

Several lines of evidence indicate that MmuPV1 shares a number of genomic, molecular and pathological features with high-risk cutaneous, beta HPVs and thus could be a useful model to study these important human pathogens. These include our observation that MmuPV1 contains separate promoters for E6 and E7, and the additional facts that MmuPV1 causes squamous cell carcinomas at cutaneous sites [57], lacks an E5 ORF [4] and encodes an E6 protein that shares with HPV8 E6 the ability to bind MAML1 and SMAD2/SMAD3 but not E6AP and p53 [58].

In this study, we also performed a careful assessment of the timing of onset of viral gene expression and productive amplification of viral DNA in the context of emerging warts. These studies used a fixed amount of virus (10^8^ VGE per infection site) applied to three scarified sites on the tail of each nude mouse. We found that both viral late gene expression (L1 RNA and capsid) and viral DNA amplification could be observed in differentiated cell compartment as early as 10 days post infection, well before overt warts can be observed, which starts at 4 weeks with this dose of virus and this strain of mouse. We observed variability in the onset of detectable infection, be it at a microscopic or overt level, with the some infection sites not showing any microscopic evidence of infection until the 4-week time point when overt warts first appeared. Interestingly, we observed a reproducible spatial pattern in which sites of infection at the base of the tail gave rise to faster growing warts than sites infected at the tip of the tail. The reason for this is unknown, but some possibilities may include blood flow, temperature, and grooming behavior.

Another feature of the time course study is that L1 positive cells were throughout the epithelium, including basal cells in mature warts harvested at 3 or 6 months post-infection, an uncommon feature of MmuPV1 observed by others [6]. However, the L1-positive cells were restricted to differentiated cells in the early time points, out to day 28 post-infection (Fig 8A), suggesting that the complete nature of the viral life cycle is realized at times later than 4 weeks post-infection. Interestingly, canine oral papillomavirus (COPV) [59] is also found to be amplified in basal epithelial cells, though the timing at which this first appears is slightly different. COPV, while a mucosal papillomavirus, is closely related to cutaneous HPVs, HPV-1 and HPV-63, that cause plantar warts [60,61]. Basal cells have been seen to support viral DNA amplification in lesions caused by HPV-1 and HPV-63 [62].

Lastly, we made the interesting observation that in experimentally infected nude mice that have developed warts at the sites of infection, other areas of the epidermis can show evidence for subclinical infections. These subclinical infections showed the same distribution pattern of viral RNA-seq reads as the experimentally infected sites with warts when the reads mapped to the reference MmuPV1 genome (Fig 1B), and showed evidence for productive infection, based upon the detection of viral DNA amplification and L1 expression (S1B Fig). This raises the intriguing possibility that subclinical infections may be common in immunodeficient or immunosuppressed contexts. In this regard, organ transplant patients are known to have an increased abundance of HPV DNA in randomly sampled hair follicles from clinically normal skin [63].

In conclusion, we observe a similar transcription pattern for MmuPV1 as observed with animal papillomaviruses and some HPVs. We believe this carefully mapped landscape of MmuPV1 transcription from MmuPV1-induced warts will provide a solid foundation for future understanding of MmuPV1 molecular biology, pathogenesis and immunology.

## Materials and methods

### MmuPV1 infection model and preparation of tissues for analyses

Immunodeficient athymic BALB/c *FoxN1*^*nu/nu*^ used in this study were obtained from Harlan (currently Envigo, Indianapolis, IN). All infected mice (6–8 weeks old at the time of infection) were housed in aseptic conditions in micro-isolator cages. Animals were handled only by designated personnel and personal protection gear was changed between cages to prevent any virus cross-contamination. Experimental infection was performed using quasivirions containing MmuPV1 synthetic genome as described previously [57,64,65]. The synthetic MmuPV1 genome (Gift from Dr. Chris Buck, NCI) is identical to the original wild type genome and has been described previously [6]. Briefly, 293FT cells (Thermo Fisher Scientific, Waltham, MA), a fast growing 293T cell line, were co-transfected with a codon optimized MmuPV1 capsid protein expression plasmid (pMusSheLL, gift from Dr. Chris Buck, NCI) [6,66] and recircularized MmuPV1 synthetic genome for encapsidation. The cells were harvested 48 h after cotranscfection and virions were purified using Optiprep (Sigma-Aldrich, St. Louis, MO) gradient centrifugation. The generated quasivirions were quantified by estimating viral genome equivalents (VGE) by comparing the amount of encapsidated viral DNA in the viral stock by Southern blot analysis using MmuPV1-specific probes, followed by quantification using ImageJ software as described previously [57]. BALB/c *FoxN1*^*nu/nu*^ mice (6–8 weeks old) were infected with 2×10^8^ VGE MmuPV1 per site after scarifying skin of tail, ear or muzzle as described previously [57,58]. The wart tissues from three anatomical sites (ear, tail and muzzle) were collected from each animal 6 months post-infection and snap-frozen in liquid nitrogen for RNA isolation and a portion of the papillomas was excised, fixed in 10% neutral buffered formalin and embedded in paraffin. Serial sections (5 μm thick) were stained with hematoxylin and eosin (H&E) and evaluated for histopathological features and processed for subsequent analyses.

### Ethics statement

All animal experiments were performed in full compliance with standards outlined in the "Guide for the Care and Use of Laboratory Animals” by the Laboratory Animal Resources (LAR) as specified by the Animal Welfare Act (AWA) and Office of Laboratory Animal Welfare (OLAW) and approved by the Governing Board of the National Research Council (NRC). Mice were housed at McArdle Laboratory Animal Care Unit in strict accordance with guidelines approved by the Association for Assessment of Laboratory Animal Care (AALAC), at the University of Wisconsin Medical School. All protocols for animal work were approved by the University of Wisconsin Medical School Institutional Animal Care and Use Committee (IACUC, Protocol number: M02478).

### MmuPV1 L1-cytokeratin dual immunofluorescence

A home-based tyramide-based signal amplification (TSA) method was developed as described previously to detect MmuPV1 L1 [67,68]. Formalin-fixed paraffin embedded tissue slides were deparaffinized after 3 changes of xylene followed by rehydration in ethanol series (100%, 95%, 70%, 50% and finally double distilled water). Endogenous peroxidase activity was blocked using 0.3% hydrogen peroxide in methanol. Antigen retrieval was performed using antigen retrieval buffer (pH = 9.0, Abcam, Cambridge, MA, #ab93684) for 20 minutes in a microwave. Slides were cooled to room temperature and blocked for 1 h at room temperature in blocking buffer (Perkin Elmer, Fermont, CA, #FP1012). Rabbit sera against MmuPV1 L1 (Gift from Dr. Chris Buck, NIH) [6,69] was diluted at 1:5000 in blocking buffer and applied to sections overnight at 4°C. Samples were incubated with goat anti-rabbit-HRP secondary antibody (at 1:500 dilution) in blocking buffer for 1 h at room temperature. Subsequently, the secondary antibody was biotinylated by incubating with biotin-tyramide (10 μg/ml) for ten minutes as described previously [68]. Slides were rinsed with PBS (phosphate-buffered saline) containing 0.1% Tween-20 and a cytokeratin cocktail containing equal amounts of anti-K14 (BioLegend, San Diego, CA, #PRB-155P) and anti-K10 (BioLegend, #PRB-159P) at 1:1000 dilution was applied at room temperature for 1 hour. Slides were rinsed with PBS containing 0.1% Tween-20 and incubated with secondary detection reagents as follows—anti-rabbit conjugated with Alexa Fluor488 (Thermo Fisher Scientific, #A11008) at 1:500 to detect K10 and K14, and Streptavidin-Alexa Fluor-594 (Thermo Fisher Scientific #S-32356) at 1:500 to detect biotinylated L1 for 1 hour at room temperature. Tissues were counter stained with Hoechst for cellular DNA and coverslips were mounted using ProLong gold antifade (Thermo Fisher Scientific, #P36930).

### Fluorescent in situ hybridization (FISH) for MmuPV1 DNA

MmuPV1 DNA FISH was performed as described previously [57,70]. This protocol has been adapted from a DNA FISH protocol used to detect Epstein Barr Virus (EBV) DNA in monolayer cells and is described in detail at: https://mcardle.oncology.wisc.edu/sugden/protocols.html. Briefly, formalin-fixed paraffin embedded tissue slides were baked at 65°C overnight and deparaffinized using xylene followed by treatment with 100% ethanol. Slides were then boiled in 10 mM sodium citrate buffer (pH = 6.0) for 30 minutes in a microwave. Slides were rinsed with PBS and completely dried before pre-hybridizing with 2 x SCC containing RNase A and 0.5% IPEGAL (pH = 7.0) for 30 min at 37°C. Slides were dehydrated using a series of ice cold ethanol (70%, 80%, 95%) for 2 min each. Slides were dried by placing them in an empty container at 50°C for 5 min and then placed in denaturation solution [28 ml formamide, 4 ml 20 x SSC (pH = 5.3) and 8 ml water] at 72°C for 2 min. The ethanol series was repeated again, and after drying the sections, denatured probe was added to the slides. A biotin-16-dUTP (Sigma-Aldrich, #11093070910) labeled probe was hybridized to tissue overnight at 37°C in a humidified chamber. To make the probe, nick translation was used to label the entire MmuPV1 plasmid DNA (pMusPV [6]) with biotin. Slides were then washed twice for 30 min with 2 x SSC and 50% formamide at 50°C followed by two washes for 30 min with 2 x SSC at 50°C. Signals were detected with streptavidin conjugated to Cyanine-3 (Sigma-Aldrich, #S6402) at 1% by volume in STM solution (4 x SSC, 5% non-fat dried milk, 0.05% Tween-20, 0.002% sodium azide) for 30 min at 37°C. Nuclei were counterstained with Hoechst coverslips were mounted using ProLong gold antifade (Thermo Fisher Scientific, #P36930).

### Image acquisition

High resolution wide-field fluorescent images were acquired by means of a super-resolution Leica SP8 STED confocal microscope equipped with a motorized stage. This microscope is equipped with PMT and HyD lasers. All images were taken by means of a 20X objective lens (Specifications: HC PL APO 20x/0.75 CS2, Dry). The images were acquired by tile-scanning by marking positions around the region of interest on the LAS-X suite (version: 2.0.1). The merged wide-field image was obtained by automatic stitching of individual styles by means of in-built auto stitching algorithm part of the LAS-X suite. All other images for tissue analyses were captured using a Zeiss AxioImager M2 microscope and AxioVision software version 4.8.2 (Jena, Germany).

### Total RNA-seq and bioinformatics analysis

The tissues were homogenized in TriPure reagent (Roche, Indianapolis, IN, #11667165001). Total RNA was extracted according to TriPure extraction protocol and treated with TURBO DNA-free Kit (Thermo Fisher Scientific, Waltham, MA, AM1907) to eliminate all traces of viral DNA. The RNA concentration and integrity were assessed by Bioanalyzer 2100 (Agilent, Santa Clara, CA). After removal of ribosomal RNA, the total RNA sequence libraries were prepared using Illumina Stranded Total RNA (Illumina, RS-122-2201, San Diego, CA) protocol with TruSeq V4 chemistry and sequenced in the Sequencing Facility of NCI on Illumina HiSeq 2500 with 2×125 nts modality and depth of 100 million reads per sample. The obtained reads were trimmed of adapters and low-quality bases and aligned to MmuPV1 reference genome (NC_014326; GI:301173443) with start site at nt 7088 using STAR aligner package [18,71]. This arrangement makes the linear MmuPV1 to end at nt 7087, approximately 186 nts from L1 stop codon. Thus, all nucleotide positions described in this report refer to the reference genome sequence (GenBank Acc. # NC_014326) [4]. Integrative Genomics Viewer (IGV, Broad Institute) program was used to visualize MmuPV1 reads coverage. The data discussed in this publication have been deposited in NCBI’s Gene Expression Omnibus and are accessible through GEO Series accession number GSE104118 (www.ncbi.nlm.nih.gov/geo/query/acc.cgi?acc=GSE104118). Additional criteria were used to identify the real splice junctions extracted by STAR aligner: (1) a threshold of median spliced read alignment overhang >10 nt; (2) the number of uniquely mapping reads crossing the junction >50 to ensure filtering out sporadic false junctions; (3) entropy of overhang length distribution >1 to filter out junctions with unevenly distributed overhangs. A Sashimi plot for splice junction visualization was generated by IGV.

### Rapid amplification of cDNA ends (RACE) and PacBio Iso-seq sequencing

The 5′ and 3′ RACE assays were carried out using a Smart RACE cDNA amplification kit (Clontech, Mountain View, CA, #634858) according to the manufacturer’s instructions using 1 μg/reaction of total RNA as template [9]. The primers used in the assays are in S1 Table (see supplemental materials). The final PCR products were gel purified, cloned into pCR2.1-TOPO vector (Thermo Fisher Scientific) and sequenced by Sanger sequencing (Macrogen USA, Rockville, MD). To obtain the comprehensive coverage of viral TSS, the 5’ RACE products obtained by Pr3299 and Pr5452 were subjected to single molecule, real-time sequencing using PacBio Iso-seq technology (Pacific Biosciences, Menlo Park, CA). The mouse papillomavirus amplicons produced using a 5’ RACE method were used as the input for PacBio Iso-seq sequencing. Using the SMRTbell Template Prep Kit 1.0 (Pacific Biosciences), the cDNA underwent damage repair, A-tailing, and A/T hairpin adaptor ligation. Following adaptor ligation, the libraries were digested with Exonuclease III and VII to remove non-ligated and nicked SMRTbell molecules. AMPure PB beads (Pacific Biosciences) were used at a 0.6 x ratio to clean up all enzymatic reactions throughout library construction. The DNA/Polymerase Binding Kit P6 v2 (Pacific Biosciences) was used for annealing the sequencing primer and binding the polymerase to the final libraries. The MagBead loading Kit (Pacific Biosciences) was used for loading the polymerase-bound SMRTbell molecules onto the SMRT Cell. Each SMRT Cell was sequenced for six hours using the DNA Sequencing Reagent Kit 4.0 v2 (Pacific Biosciences) on a PacBio RS II sequencer. The obtained sequence reads were trimmed of adaptors and only the reads containing a specific adaptor at 5’ end introduced by 5’RACE were considered the full-length and used for mapping to MmuPV1 reference genome in further analysis and visualized in the IGV program. The position of 5’ends of the full-length reads were extracted and quantified in promotor usage analysis.

PacBio SMRT Analysis Package (smrtpipe.py v1.87, with default settings) was used to process raw data into circular consensus sequence (CCS) for further analyses. First, the raw data was processed into error corrected reads of insert (ROI’s) by RS_Read Of Interest (ROI).1 protocol provided in the SMRT Analysis Package. The ROI’s were then processed using the Classify module with default parameters to remove adapter sequences, poly (A) tails, artificial chimeras, and 3′ truncated transcript sequences which resulted into full-length non-chimeric (FLNC) reads by RS_IsoSeq.1 protocol. For further analyses, we mapped the CCS reads and FLNC reads into the MmuPV1 genome and identified the transcription start sites by BLAT (with-fine option otherwise default options) [72]. Additional computational analyses were performed with Python (version 3.5, https://www.python.org/).

### RT-PCR

To remove contaminated genomic DNA, the total RNA was treated with TURBO DNA-free Kit (Thermo Fisher Scientific). Reverse transcription (RT) was performed with the SuperScript II kit (Thermo Fisher Scientific, #11904–018). Amplification of reversed transcribed cDNA was performed by PCR using the Platinum SuperFi Taq Polymerase Kit (Thermo Fisher Scientific, #12351–050) according to the manufacturer’s protocols. The MmuPV1-specific primers (S1 Table, see supplemental material) were used to detect viral transcripts. GAPDH RNA served as a sample loading control by using a mouse GAPDH-specific primer pair (forward oMA1, 5’-ATGTTCCAGTATGACTCCAC-3’ and backward oMA2, 5’-TGACAATCTTGAGTGAGTTG-3’). All PCR amplifications were performed under same conditions: on a primary denaturation step at 94°C for 2 min, followed by 25 or 35 cycles of 30 sec at 94°C, 45 sec at 55°C and 60 sec at 72°C, and final extension for 10 min at 72°C.

### Northern blot analysis

Total RNA used for Northern blot analysis was isolated from mouse ears with or without MmuPV1 infection or extracted from regular HEK293 cells (ATCC, Manassas, VA) transfected with individual MmuPV1 ORF expression vectors. Total RNA from mouse ears without MmuPV1 subclinical infection served as a MmuPV1-negative RNA control and was pooled RNA isolated from ears of two naïve, freshly arrived female mice from Harlan lab in ~4 months of age, with no detectable MmuPV1 reads by RNA-seq analysis. In general, total 5 μg of RNA from each sample was mixed with NorthernMax Formaldehyde loading dye (Thermo Fisher Scientific, #AM8552)) and denatured at 75°C for 15 min. The RNA samples were then separated in 1% (wt/vol) formaldehyde-containing agarose gels in 1× morpholinepropanesulfonic acid (MOPS) running buffer, transferred onto a GeneScreen Plus hybridization transfer membrane (Perkin Elmer, Waltham, MA, #NEF987001PK) and UV light crosslinked and stained by ethidium bromide for 18S ribosome level as an internal loading control. The membrane was then prehybridized with PerfectHyb Plus hybridization buffer (Sigma-Aldrich, #H7003) for 2 h at 42°C followed by overnight hybridization with a MmuPV1-specific oligo probe as described [73]. MmuPV1-specific oligo probes and a U6-specific probe (oST 197, 5’-AAAATATGGAACGCTTCACGA-3’) were prepared by end-labeling of antisense oligos (S1 Table) with γ-^32^P using T4 PNK (Thermo Fisher Scientific, #18004–010). After hybridization, the membrane was washed once with a 2× SSPE (1× SSPE: 0.18 M NaCl, 10 mM NaH_2_PO_4_ and 1 mM EDTA [pH 7.7])-0.1% SDS solution for 5 min at room temperature and twice with 0.1× SSPE-0.1% SDS for 15 min at 42° and then exposed to a PhosphorImager screen and X-ray film.

### RNase protection assay (RPA)

The radioactive RNA probes were prepared by *in vitro* transcription in the presence of [α-^32^P]CTP with Riboprobe System-T7 (Promega, Madison, WI, #P1446), using PCR products with a built-in T7 promoter as DNA templates. The following primers were used for MmuPV-1 DNA template preparation: oXYX-12 and oXYX-23 (S1 Table). The RNase protection assay (RPA) was performed with an RPA III kit (Ambion, Austin, TX, #1414) according to the manufacturer’s instructions with minor modifications. Briefly, 4 ng of each probe (specific activity, 35,000 cpm/ng) was hybridized overnight at 50°C with 30 μg of total tissue RNA in hybridization buffer and then digested with an RNase A-T1 mixture for 30 min at 37°C. Five micrograms of yeast RNA was used as a negative control (MmuPV1 -). Protected RNA fragments were separated in a denaturing 8% polyacrylamide gel containing 8 M urea. MmuPV-1 DNA sequencing ladders generated with the ^32^P-labeled Primer Pr7237 (oXYX-28) (S1 Table) were used as size markers and run along with the RPA products as described [73]. Autoradiographic data were captured with a Typhoon Imaging System (GE Healthcare Life Sciences, Pittsburgh, PA) and analyzed with ImageQuant software (GE Healthcare Life Sciences).

### RNA-ISH

For in situ detection of viral transcripts, the tissues were fixed in 10% neutral buffer formalin for 20 h at room temperature, dehydrated, and embedded in paraffin. The sections were cut into 5 μm slides and subjected to RNA-ISH using RNAscope technology (Advanced Cell Diagnostics, Newark, CA) as recommended by manufacturer. Two custom designed probes derived from MmuPV1 genome were used: E1^E4 (nt 3139–3419) and L1 (nt 5372–6901). The signal was detected by colorimetric staining using RNAscope 2.5 HD Assay—BROWN followed by hematoxylin Gill’s No. 1 solution (Sigma-Aldrich, #GHS116) counterstaining. The slides were dehydrated, mounted in Cytoseal XYL (Thermo Scientific, #8312–4), and scanned at 40× resolution using Aperio CS2 Digital Pathology Scanner (Leica Biosystem, Buffalo Grove, IL). To distinguish viral RNA signal from viral genomic DNA signal, the MmuPV1-infected tissue sections with or without pre-treatment with DNase I or both DNase I and RNase A/T1 were compared in parallel in the RNAscope assays. To carry out DNase I or RNase treatment, all tissue sections after rehydration were digested first with RNAscope Protease Plus for 30 min and then followed by 20 units of DNase I (Thermo Fisher Scientific, cat. No. #EN0521) diluted in 1 x reaction buffer with MgCl_2_ for 30 min at 40°C or by 20 units of DNase I and 500 ug of RNase A (Qiagen, #1006657) plus 2000 units of RNase T1 (Fermentas, Waltham, MA, #EN0542) diluted in 1 x reaction buffer with MgCl_2_ for 30 min at 40°C.

### MmuPV1 ORF cloning and expression analysis

To express viral proteins, the cDNAs of individual ORF under the optimized Kozak context were amplified by RT-PCR from total RNA isolated from infected tissues and cloned into pFLAG-CMV-5.1 (Sigma-Aldrich) vector in frame with a C-terminal FLAG tag. The obtained plasmid DNA (2 μg) was utilized to transfect HEK293 cells (2.5 × 10^5^) plated in a 12-well plate using LipoD293 transfection reagent (SignaGen Laboratories, Rockville, MD, #SL100668). In some cases the cells were treated 24 h after transfection with 10 μM proteasome inhibitor MG132 (Sigma-Aldrich, #474790) for 6 h. The primary mouse keratinocytes were cultivated as described [74] in the presence of Rho kinase inhibitor Y-27632 (Enzo Life Sciences, Farmingdale, NY, #ALX-270-333). The mouse keratinocytes (1 × 10^5^) were transfected with 1μg of plasmid DNA using Amaxa P3 Primary Cells 4D Nucleofector X Kit S (Lonza, Walkersville, MD, #V4XP-3032) and program DS-138 as recommended by manufacturer. After transfection, the keratinocytes were plated into 24-well plate containing mitomycin-treated feeder 3T3 cells and incubated for 45 hours in the absence of Rho kinase inhibitor.

Total protein extracts and total RNA were prepared 24 h after transfection of HEK293 cells and the expressed individual viral proteins were determined by Western blotting with a rabbit polyclonal anti-FLAG antibody (Sigma-Aldrich, #F7425). Total RNA was resolved on a 1% formaldehyde-agarose gel, stained by ethidium bromide for 18S ribosomal RNA as a sample loading control, and examined by Northern blotting for individual viral gene transcripts expressed from the transfected plasmid by a γ-^32^P-labeled oligo probe (oVM79, 5’-GGGCACTGGAGTGGCAAC-3’) which hybridizes to a common 3’ UTR region downstream of the FLAG-tag, but upstream of the poly(A) site. U6 snRNA served as a loading control and was detected using a γ-^32^P-labeled, U6-specific oligo probe oST197.

### Immunofluorescent staining to detect FLAG-tagged viral proteins in cultured cells

HeLa cells (2.5 × 10^5^, ATCC) growing on the coverslips were transfected with each vector (0.5–1 μg) encoding a FLAG-tagged viral protein by using LipoD293 transfection reagent (SignaGen Laboratories). The cells at 24 h after transfection were fixed, permeabilized, and stained with a monoclonal anti-FLAG M2 antibody (Sigma-Aldrich, # F1804) in combination with Alexa Flour488-labeled anti-mouse secondary antibody (Thermo Fisher Scientific, #A11029) as described before [75,76]. The cell nuclei were counterstained by Hoechst 33342 dye (Thermo Fisher Scientific, #H3570).

## Supporting information

S1 Fig Evidence of MmuPV1 infection of *FoxN1*^*nu/nu*^ mice. (A) Representative wart arising on the snout and ear of *FoxN1*^*nu/nu*^ mice from which RNA was isolated and analyzed for this study. All papillomas were analyzed by H&E staining, L1 (red)-Krt (Krt10 and Krt14, green) immunofluorescence and MmuPV1 DNA FISH (red). For both IF and FISH nuclei were counter-stained with Hoechst for host cell DNA. (B) Ears of *FoxN1*^*nu/nu*^ mice MmuPV1-induced tail warts show evidence for subclinical infection. Tissue section images of a tail wart (top panels) and experimentally uninfected ear from the same animal (bottom panels) were stained for MmuPV1 L1 protein by IF (left panels—red) and MmuPV1 DNA by FISH (right panels—red). Noted evidence for L1 expression and viral DNA amplification in bottom panels is indicative of subclinical infection.
(TIF)

S2 Fig Gel profiles of 5’ RACE products from individual RACE primers and their mapped TSS. (A) Agarose gel electrophoresis of 5’ RACE products amplified by the indicated primers (Fig 2A) from the MmuPV1-infected tissue total RNA. Each number on the gel indicates the designated RACE product (band) being purified, cloned and sequenced. (B) Mapped TSS from each 5’RACE product by TA cloning and Sanger sequencing. Each TSS from the corresponding RACE product (band) in (A) was mapped to the indicated nucleotide position in the MmuPV1 genome according to its frequency from the screened colonies. Numbers in parenthesis indicate how many colonies contain the mapped TSS among the screened colonies (see details for all mapped TSS in S2 Table). *, TSS mapped by direct sequencing of the gel-purified product (band).
(TIF)

S3 Fig Mapped TSS and their surrounding nucleotide sequence in the MmuPV1 genome. (A) IGV profiles of the mapped TSS by 5’RACE in combination with PacBio Iso-seq. Viral primers Pr3299 (Blue) from the early region and Pr5452 (red) from the late region of MmuPV1 genome were used for 5’RACE and PacBio Iso-seq on the wart-tissue-derived total RNA (see Fig 2 for more details). Arrow indicates the nucleotide position with a highest number of PacBio Iso-seq reads as the mapped TSS (also designated as a promoter (P) start site) in the MmuPV1 genome. The scales in upper right corner shows the reads coverage depth set to autoscale. (B) The sequence profiles of mapped TSS by 5’ RACE in combination with TA cloning-Sanger sequencing. The arrows above sequences mark the most prevalent TSS mapped by an indicated primer used for each 5’ RACE, TA cloning and Sanger sequencing). See all analyzed colonies derived from the corresponding 5’ RACE products from S2 Table. Only the sequence containing a 5’RACE adaptor sequence was considered as the full-length RACE product. The grey boxes represent a predicted “TATA” boxes with an arrow indicating the nucleotide position in the MmuPV1 genome.
(TIF)

S4 Fig Confirmation of the mapped MmuPV1 late transcription start site at nt 7107 and the mapped late polyadenylation cleavage site at nt 7063 using RNase protection assay (RPA). RPA was performed on 30 μg of total RNA from MmuPV1-infected ear lesions (MmuPV1 +) or 5 μg of yeast tRNA (MmuPV1 -) with 4 ng of a ^32^P-labeled antisense RNA probe covering MmuPV1 genome nt 6846 to 7237 which was prepared by in vitro transcription. The same PCR template used for in vitro transcription was also cloned into the pCR2.1 vector (Invitrogen) and served as a template for sequencing with a ^32^P-labeled primer Pr7237 (oXYX-28). The protected products were separated along with sequencing ladders (A, G, C and T) and 10-bp and 100-bp DNA ladders on an 8% denaturing polyacrylamide gel. The arrow on the gel indicates the mapped TSS for the protected RPA product along with its corresponding sequence (bolded red) on the gel right. The arrowhead (▲) indicates the protected RPA product derived from usage of the mapped late polyadenylation cleavage site at nt7063.
(TIF)

S5 Fig Identification of a minor polyadenylation site in the L1 coding region. (A) A diagram of MmuPV1 genome with major ORFs and long control region (LCR). An arrow indicates the primer (Pr5433) located in the L1 coding region used for 3’RACE on total RNA isolated from MmuPV1-infected wart tissue. (B) The size of 3’RACE products obtained with Pr5433 as determined by agarose gel electrophoresis. The large and abundant 3’ RACE product (~1.63-kb) was polyadenylated at nt 7063 cleavage site (CS) and the smaller and less abundant 3’ RACE product (~194-bp) was polyadenylated at nt 5627 CS by sequencing. (C) The individual 3’RACE products were cloned into pCR2.1-TOPO vector and the insert sequence composition was determined by Sanger sequencing. Above is the reference sequence of MmuPV1 with the underlined polyadenylation signal (PAS) and the identified minor polyadenylation CS. Below is a sequence chromatograph with PAS and CS site marked by an arrow. Additional non-templated “T” before poly (A) tail was introduced by oligo-dT adaptor used of 3’RACE amplification.
(TIF)

S6 Fig Tissue expression profile of the detected E1^E4 and L1 transcripts in MmuPV1-induced warts and their differentiation from the replicated viral genomic DNA by RNAscope analysis. (A) Diagram of viral genome with major open reading frames. The incomplete ^4 (blue) and L1 (red) coding regions were used to design specific RNAscope antisense probes (arrows). (B and C) Detection of viral RNA transcripts in MmuPV1-infected ear (B) and tail (C) tissues. The bound probes were detected using DAB chromogenic staining (brown) and the slides were counterstained with Gill’s Hematoxylin solution. Ear and tail tissues were collected after 28 days of virus inoculation. (B) Expression of E1^E4 and L1 RNA transcripts in highly proliferated and differentiated keratinocytes of infected skin tissues. (C) Comparison of E1^E4 with L1 expression in a pre-tumor lesion (a) and tumor lesion (b) of infected tail tissues. The presence of many koilocytes is also a hallmark of papillomavirus infection of which the keratinocytes with active virus replication character owl’s eye appearance resulting from the nucleus compression and halo formation around it. (D and E) Differentiation of viral RNA transcripts from the replicated viral genomic DNA in MmuPV1-infected ear tissues by RNAscope RNA ISH analysis. MmuPV1-infected ear tissues with or without pre-treatment by DNase I or both DNase I and RNase A/T1 were hybridized by a MmuPV1 E4 or L1 antisense probe and examined by RNAscope RNA ISH technology (D). Note, DNase I treatment led to remove the viral DNA signal from the nucleus. Alternatively, MmuPV1-infected ear tissues with or without pre-treatment by RNase A/T1 were hybridized by a MmuPV1 E4 probe and examined by RNAscope RNA ISH technology (E). Note, RNase treatment led to remove the viral RNA signal from the cytoplasm, but remained the viral DNA signal in the nucleus.
(PDF)

S7 Fig High-resolution wide-field image representing FISH detection of amplified viral DNA (red) from the infected tail tissues. Nuclei (blue) were counterstained with Hoechst. See other details in Fig 8A. Scale bar = 100 μm.
(TIF)

S8 Fig L1 protein staining of three skin sites on the mouse tail infected with MmuPV1. Each panel consists of a high-resolution wide-field image representing immunofluorescence detection of protein L1 (red) and keratins (Krt10 & Krt14, green) in the infected site accompanied by an inset showing a high magnification on the right. Scale bar = 100 μm. All sites at 28 days post-infection show clear evidence of papillomatosis accompanied with L1 protein expression. However, papillomas grew at different rates.
(TIF)

S9 Fig Expression of MmuPV1 E1, E1^E4, L1 and L2 in mouse epithelial keratinocytes and individual MmuPV1 ORF RNAs in HEK293 cells. (A) Expression of MmuPV1 E1, E1^E4, L1 and L2 protein in mouse epithelial keratinocytes (MEK). MEK at 1 x 10^5^ in a 6-well plate were transfected with 1 μg of individual vectors expressing the indicated MmuPV1 protein as a Flag-fusion or with an empty vector pFLAG-CMV-5.1 (Sigma-Aldrich) as a negative control. Cell lysates prepared at 45 h after transfection were blotted with an anti-Flag antibody. Tubulin served as a loading control. (B) Expression level of individual MmuPV1 ORF RNAs in HEK293 cells. Total RNA (~5 μg) prepared 24 h after transfection of HEK293 (5 x 10^5^ cells per well) in a 6-well plate with 2 ug of individual plasmid DNA were examined by Northern blot with a ^32^P-labeled oligo probe (oVM79) hybridizing to a common 3’ UTR region as diagramed on the panel top. Empty vector was used as a negative control and U6 served as a sample RNA loading control. See Fig 9A for size estimation of individual ORF mRNAs. *, spliced E1 RNA.
(TIF)

S1 Table Oligo primers used in this study. (XLSX)

S2 Table Heterogeneity of the mapped transcription start sites by single colony sequencing of 5’ RACE products after TA cloning. (XLSX)

S3 Table Frequency of exon-exon splicing junction reads in individual mouse samples. (XLSX)
