# Supplementary figures and images for: The full transcription map of mouse papillomavirus type 1 (MmuPV1) in mouse wart tissues
Source: PLoS Pathog. 2017 Nov 27;13(11):e1006715. doi: 10.1371/journal.ppat.1006715 (PMC5720830; doi:10.1371/journal.ppat.1006715)

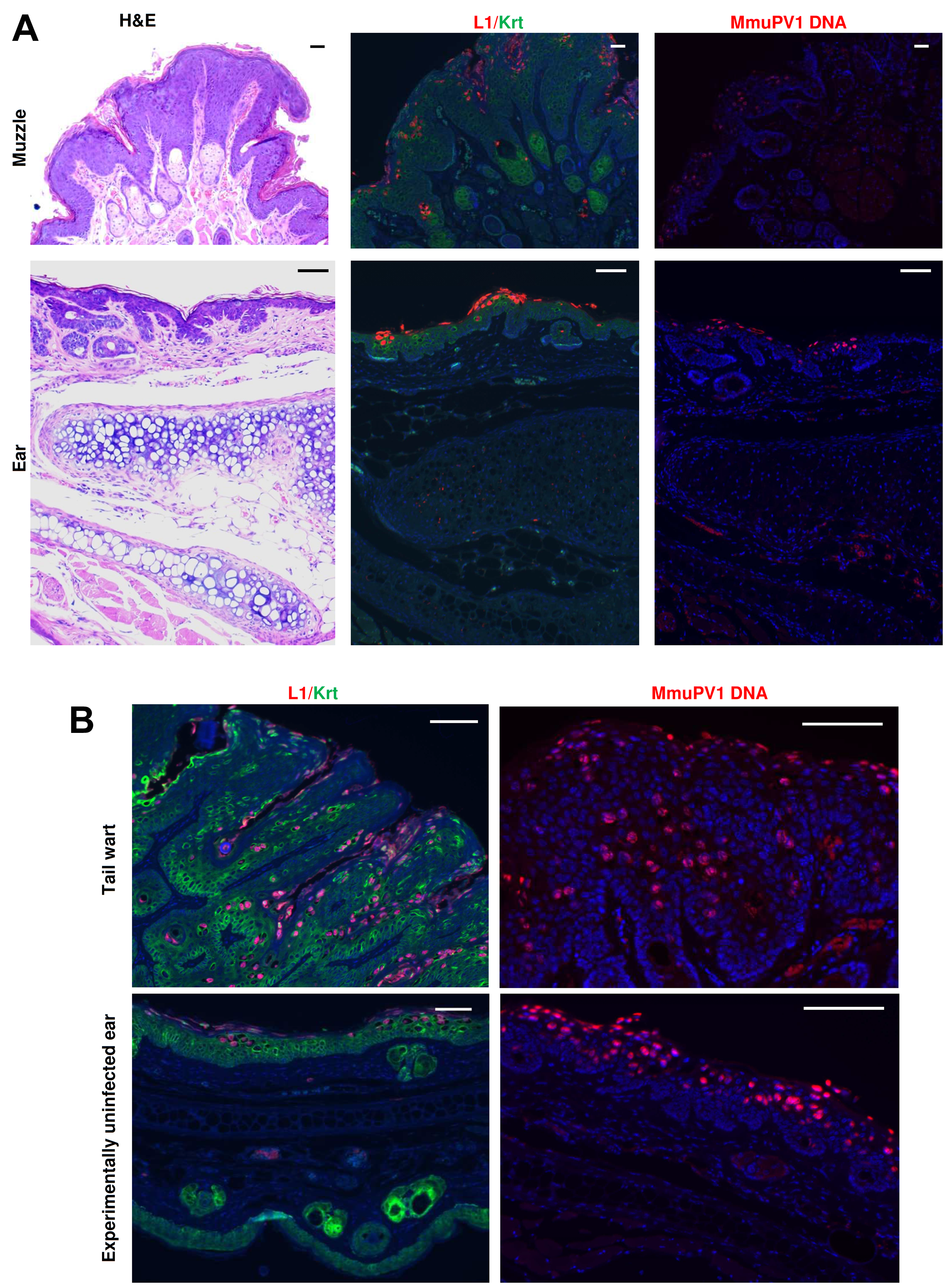

Supplement: S1 Fig — (A) Representative wart arising on the snout and ear of FoxN1nu/nu mice from which RNA was isolated and analyzed for this study. All papillomas were analyzed by H&E staining, L1 (red)-Krt (Krt10 and Krt14, green) immunofluorescence and MmuPV1 DNA FISH (red). For both IF and FISH nuclei were counter-stained with Hoechst for host cell DNA. (B) Ears of FoxN1nu/nu mice MmuPV1-induced tail warts show evidence for subclinical infection. Tissue section images of a tail wart (top panels) and experimentally uninfected ear from the same animal (bottom panels) were stained for MmuPV1 L1 protein by IF (left panels—red) and MmuPV1 DNA by FISH (right panels—red). Noted evidence for L1 expression and viral DNA amplification in bottom panels is indicative of subclinical infection. (TIF) [file ppat.1006715.s001.tif]

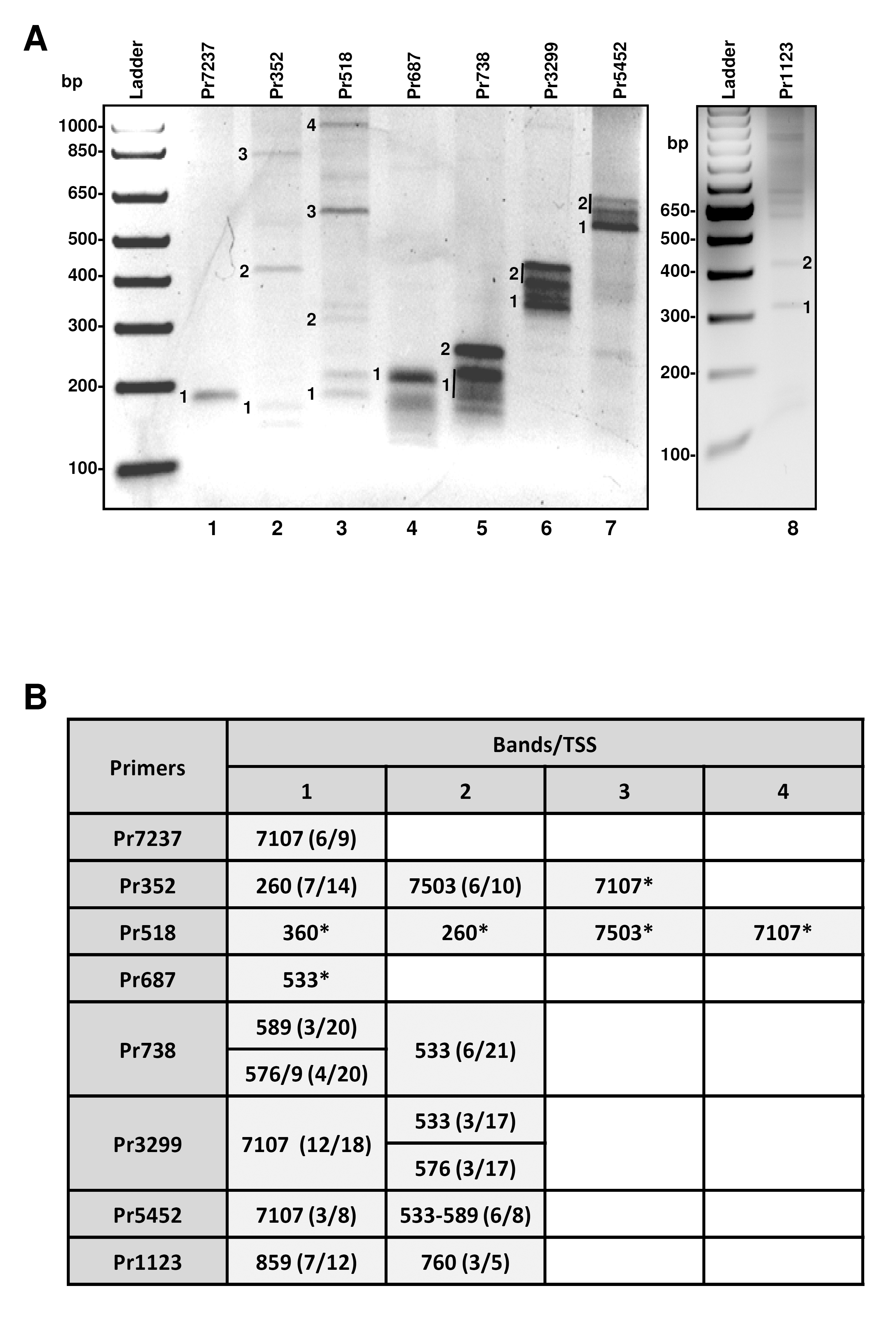

Supplement: S2 Fig — (A) Agarose gel electrophoresis of 5’ RACE products amplified by the indicated primers (Fig 2A) from the MmuPV1-infected tissue total RNA. Each number on the gel indicates the designated RACE product (band) being purified, cloned and sequenced. (B) Mapped TSS from each 5’RACE product by TA cloning and Sanger sequencing. Each TSS from the corresponding RACE product (band) in (A) was mapped to the indicated nucleotide position in the MmuPV1 genome according to its frequency from the screened colonies. Numbers in parenthesis indicate how many colonies contain the mapped TSS among the screened colonies (see details for all mapped TSS in S2 Table). *, TSS mapped by direct sequencing of the gel-purified product (band). (TIF) [file ppat.1006715.s002.tif]

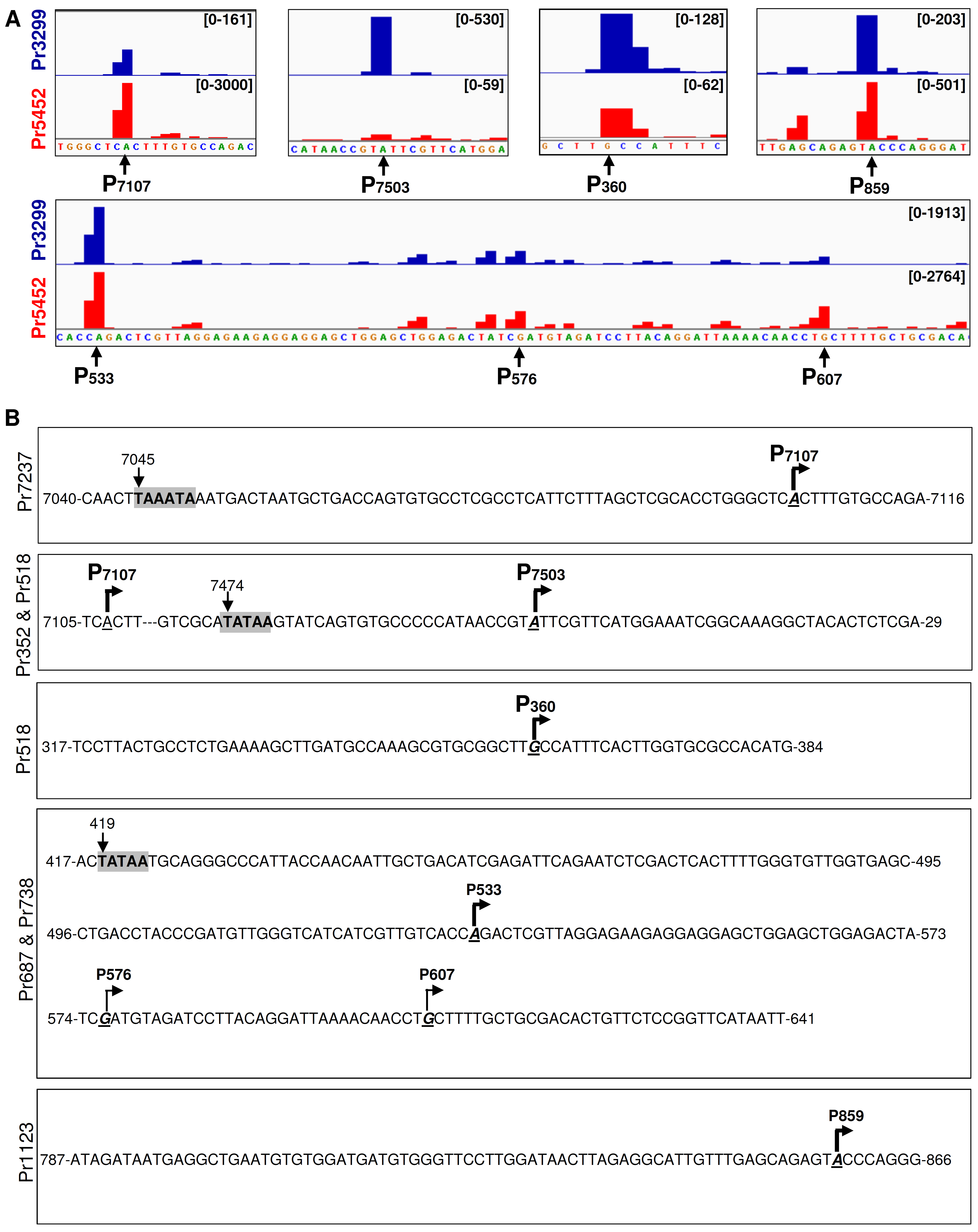

Supplement: S3 Fig — (A) IGV profiles of the mapped TSS by 5’RACE in combination with PacBio Iso-seq. Viral primers Pr3299 (Blue) from the early region and Pr5452 (red) from the late region of MmuPV1 genome were used for 5’RACE and PacBio Iso-seq on the wart-tissue-derived total RNA (see Fig 2 for more details). Arrow indicates the nucleotide position with a highest number of PacBio Iso-seq reads as the mapped TSS (also designated as a promoter (P) start site) in the MmuPV1 genome. The scales in upper right corner shows the reads coverage depth set to autoscale. (B) The sequence profiles of mapped TSS by 5’ RACE in combination with TA cloning-Sanger sequencing. The arrows above sequences mark the most prevalent TSS mapped by an indicated primer used for each 5’ RACE, TA cloning and Sanger sequencing). See all analyzed colonies derived from the corresponding 5’ RACE products from S2 Table. Only the sequence containing a 5’RACE adaptor sequence was considered as the full-length RACE product. The grey boxes represent a predicted “TATA” boxes with an arrow indicating the nucleotide position in the MmuPV1 genome. (TIF) [file ppat.1006715.s003.tif]

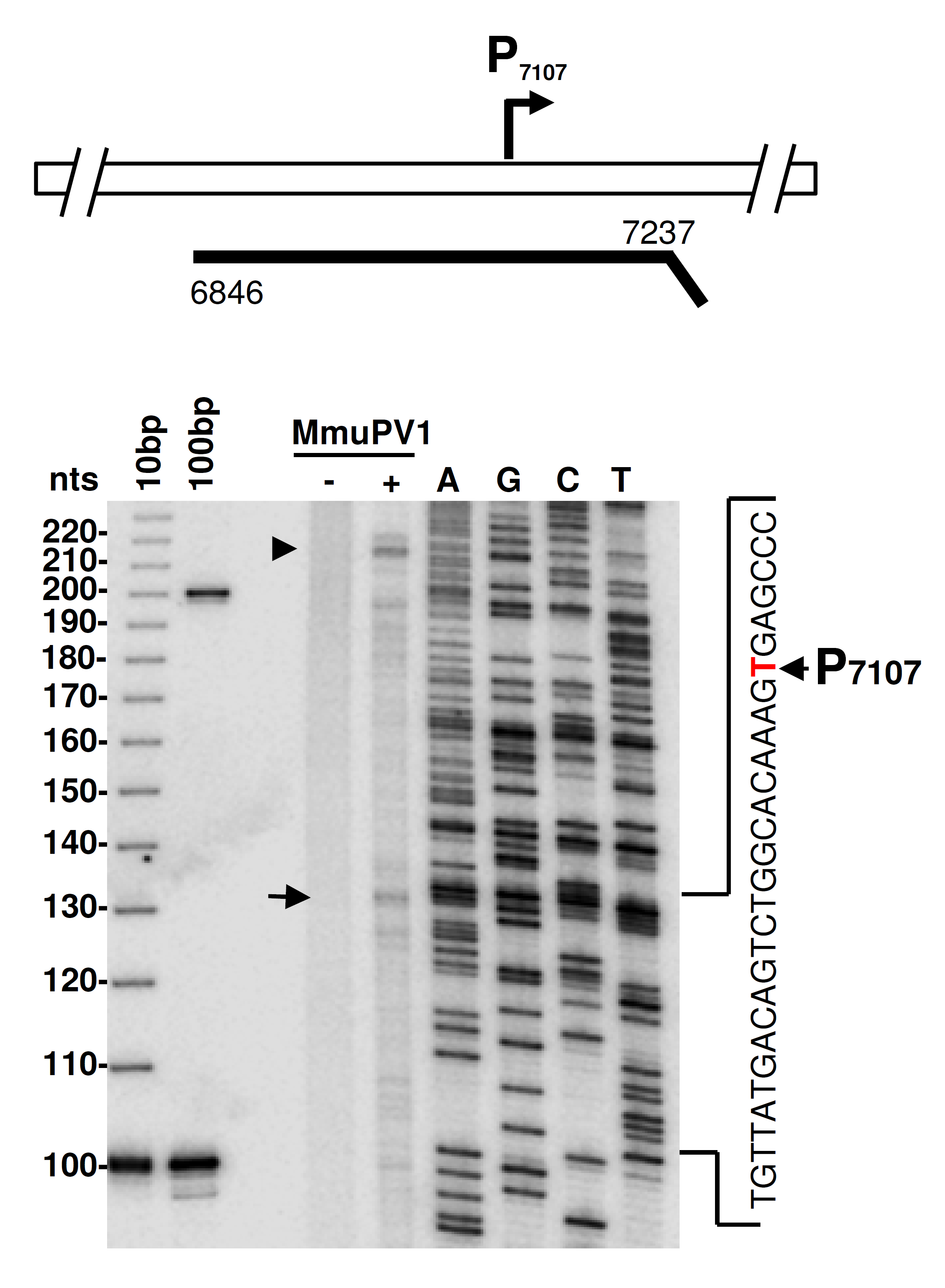

Supplement: S4 Fig — RPA was performed on 30 μg of total RNA from MmuPV1-infected ear lesions (MmuPV1 +) or 5 μg of yeast tRNA (MmuPV1 -) with 4 ng of a 32P-labeled antisense RNA probe covering MmuPV1 genome nt 6846 to 7237 which was prepared by in vitro transcription. The same PCR template used for in vitro transcription was also cloned into the pCR2.1 vector (Invitrogen) and served as a template for sequencing with a 32P-labeled primer Pr7237 (oXYX-28). The protected products were separated along with sequencing ladders (A, G, C and T) and 10-bp and 100-bp DNA ladders on an 8% denaturing polyacrylamide gel. The arrow on the gel indicates the mapped TSS for the protected RPA product along with its corresponding sequence (bolded red) on the gel right. The arrowhead (▲) indicates the protected RPA product derived from usage of the mapped late polyadenylation cleavage site at nt7063. (TIF) [file ppat.1006715.s004.tif]

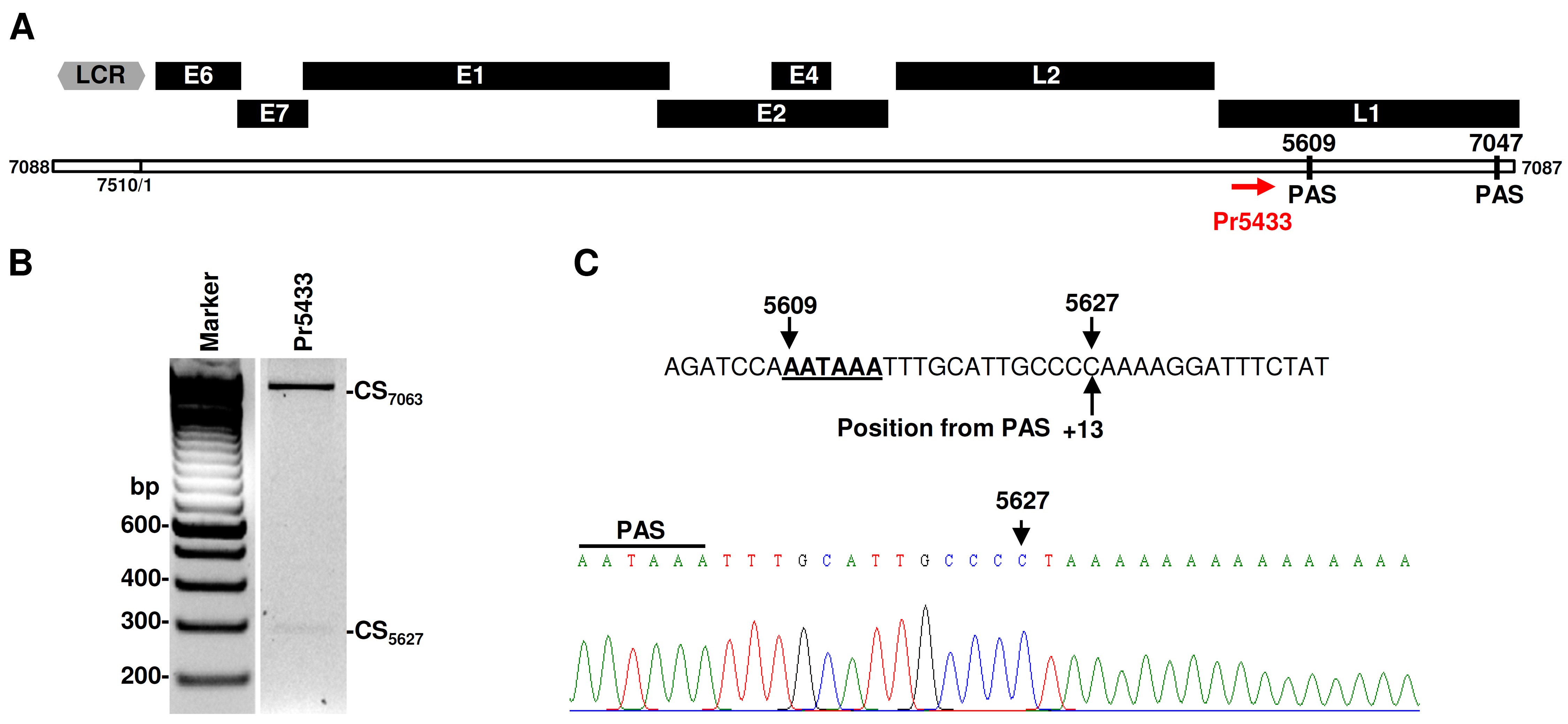

Supplement: S5 Fig — (A) A diagram of MmuPV1 genome with major ORFs and long control region (LCR). An arrow indicates the primer (Pr5433) located in the L1 coding region used for 3’RACE on total RNA isolated from MmuPV1-infected wart tissue. (B) The size of 3’RACE products obtained with Pr5433 as determined by agarose gel electrophoresis. The large and abundant 3’ RACE product (~1.63-kb) was polyadenylated at nt 7063 cleavage site (CS) and the smaller and less abundant 3’ RACE product (~194-bp) was polyadenylated at nt 5627 CS by sequencing. (C) The individual 3’RACE products were cloned into pCR2.1-TOPO vector and the insert sequence composition was determined by Sanger sequencing. Above is the reference sequence of MmuPV1 with the underlined polyadenylation signal (PAS) and the identified minor polyadenylation CS. Below is a sequence chromatograph with PAS and CS site marked by an arrow. Additional non-templated “T” before poly (A) tail was introduced by oligo-dT adaptor used of 3’RACE amplification. (TIF) [file ppat.1006715.s005.tif]

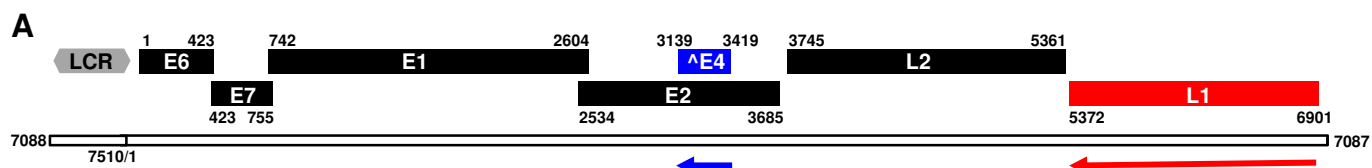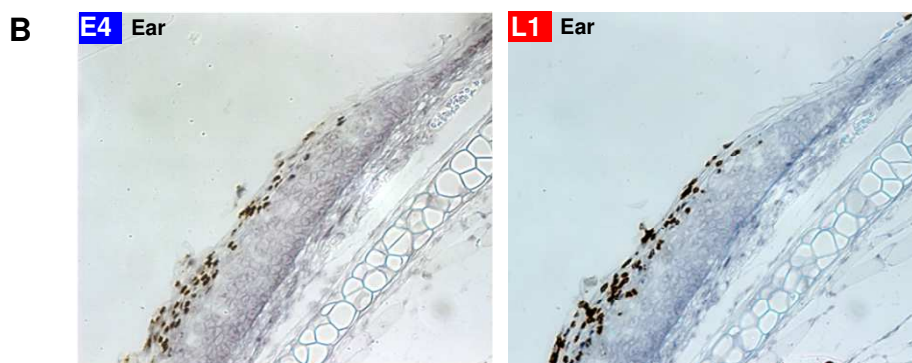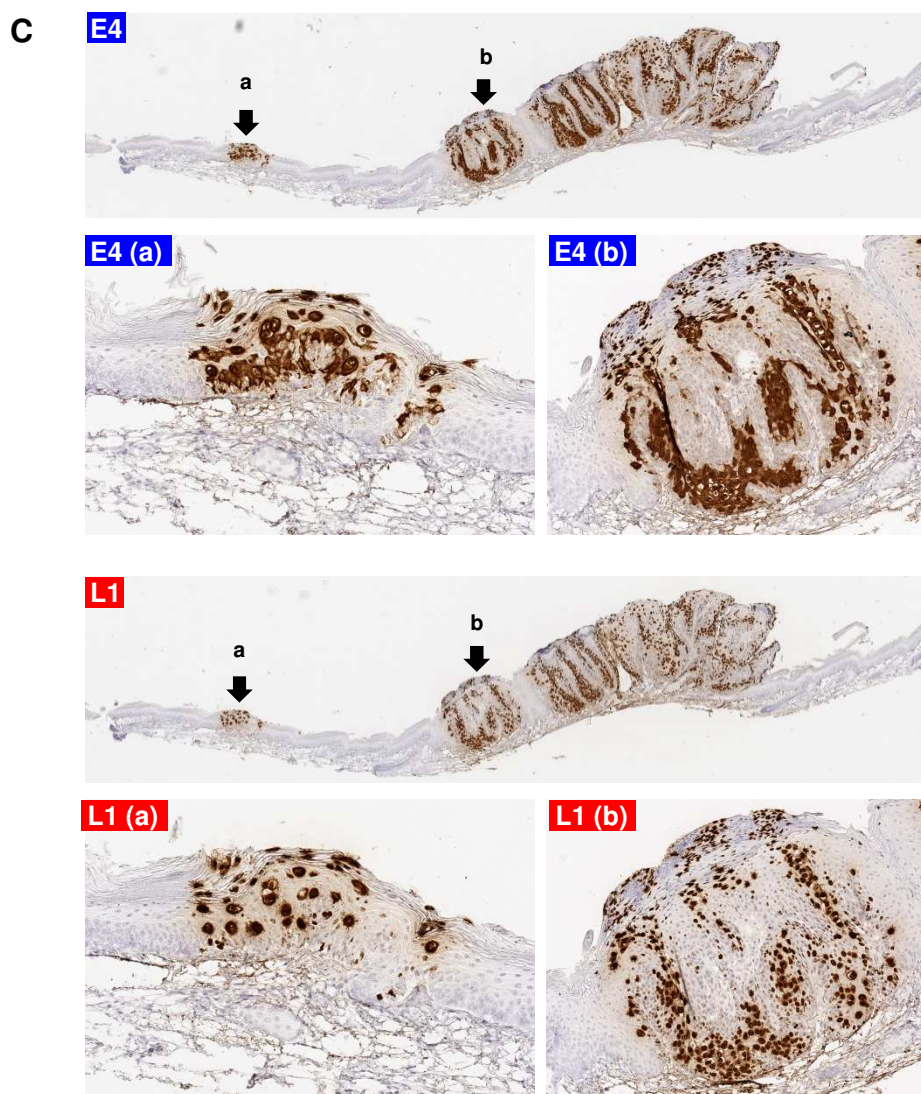

**D**

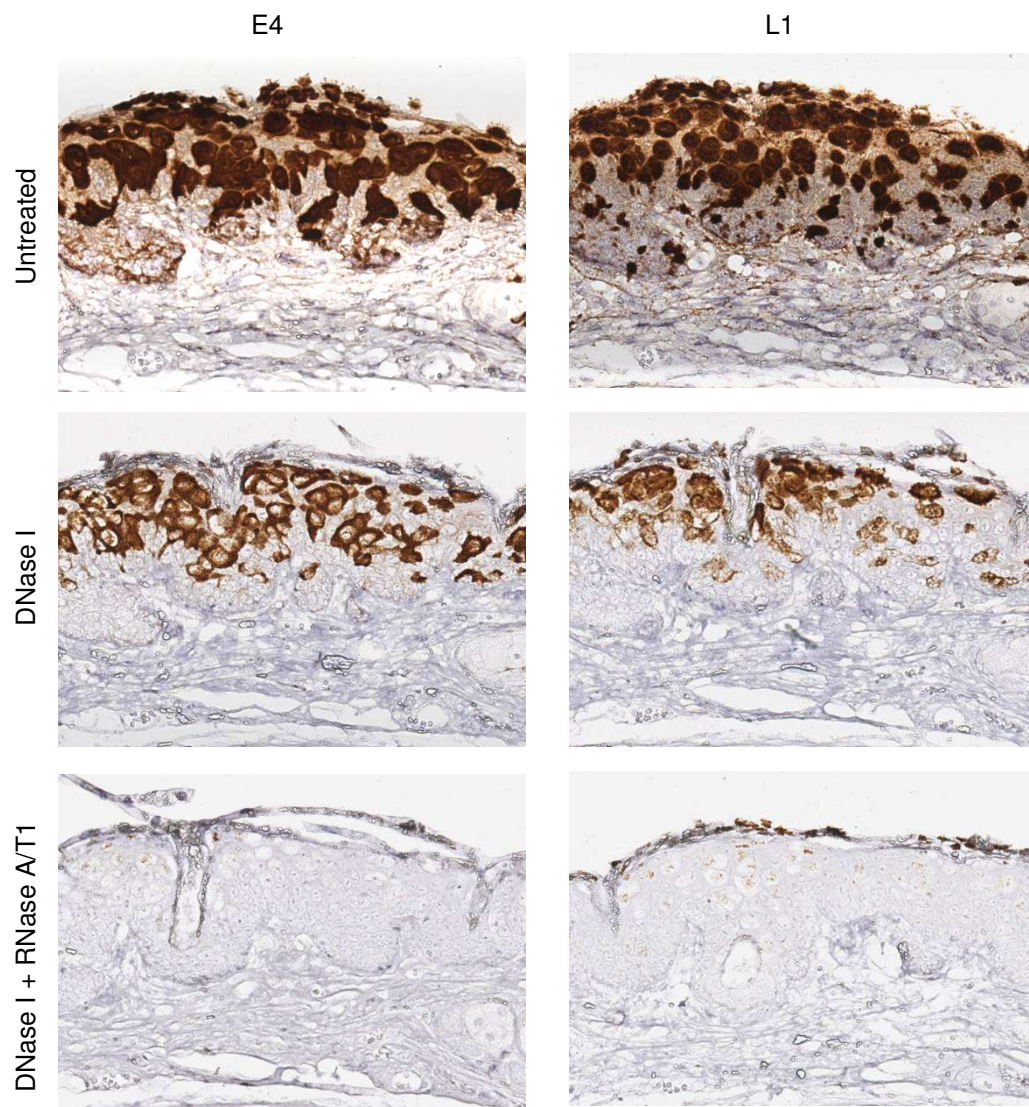

**E**

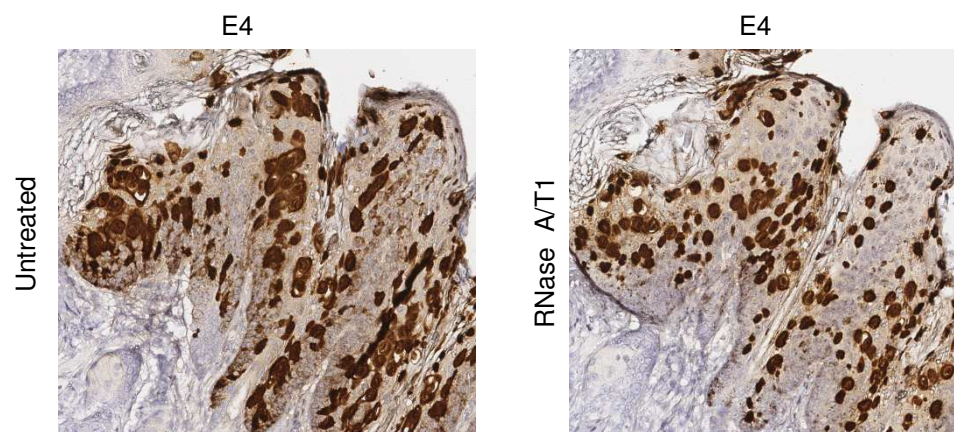

Supplement: S6 Fig — (A) Diagram of viral genome with major open reading frames. The incomplete ^4 (blue) and L1 (red) coding regions were used to design specific RNAscope antisense probes (arrows). (B and C) Detection of viral RNA transcripts in MmuPV1-infected ear (B) and tail (C) tissues. The bound probes were detected using DAB chromogenic staining (brown) and the slides were counterstained with Gill’s Hematoxylin solution. Ear and tail tissues were collected after 28 days of virus inoculation. (B) Expression of E1^E4 and L1 RNA transcripts in highly proliferated and differentiated keratinocytes of infected skin tissues. (C) Comparison of E1^E4 with L1 expression in a pre-tumor lesion (a) and tumor lesion (b) of infected tail tissues. The presence of many koilocytes is also a hallmark of papillomavirus infection of which the keratinocytes with active virus replication character owl’s eye appearance resulting from the nucleus compression and halo formation around it. (D and E) Differentiation of viral RNA transcripts from the replicated viral genomic DNA in MmuPV1-infected ear tissues by RNAscope RNA ISH analysis. MmuPV1-infected ear tissues with or without pre-treatment by DNase I or both DNase I and RNase A/T1 were hybridized by a MmuPV1 E4 or L1 antisense probe and examined by RNAscope RNA ISH technology (D). Note, DNase I treatment led to remove the viral DNA signal from the nucleus. Alternatively, MmuPV1-infected ear tissues with or without pre-treatment by RNase A/T1 were hybridized by a MmuPV1 E4 probe and examined by RNAscope RNA ISH technology (E). Note, RNase treatment led to remove the viral RNA signal from the cytoplasm, but remained the viral DNA signal in the nucleus. (PDF) [file ppat.1006715.s006.pdf]

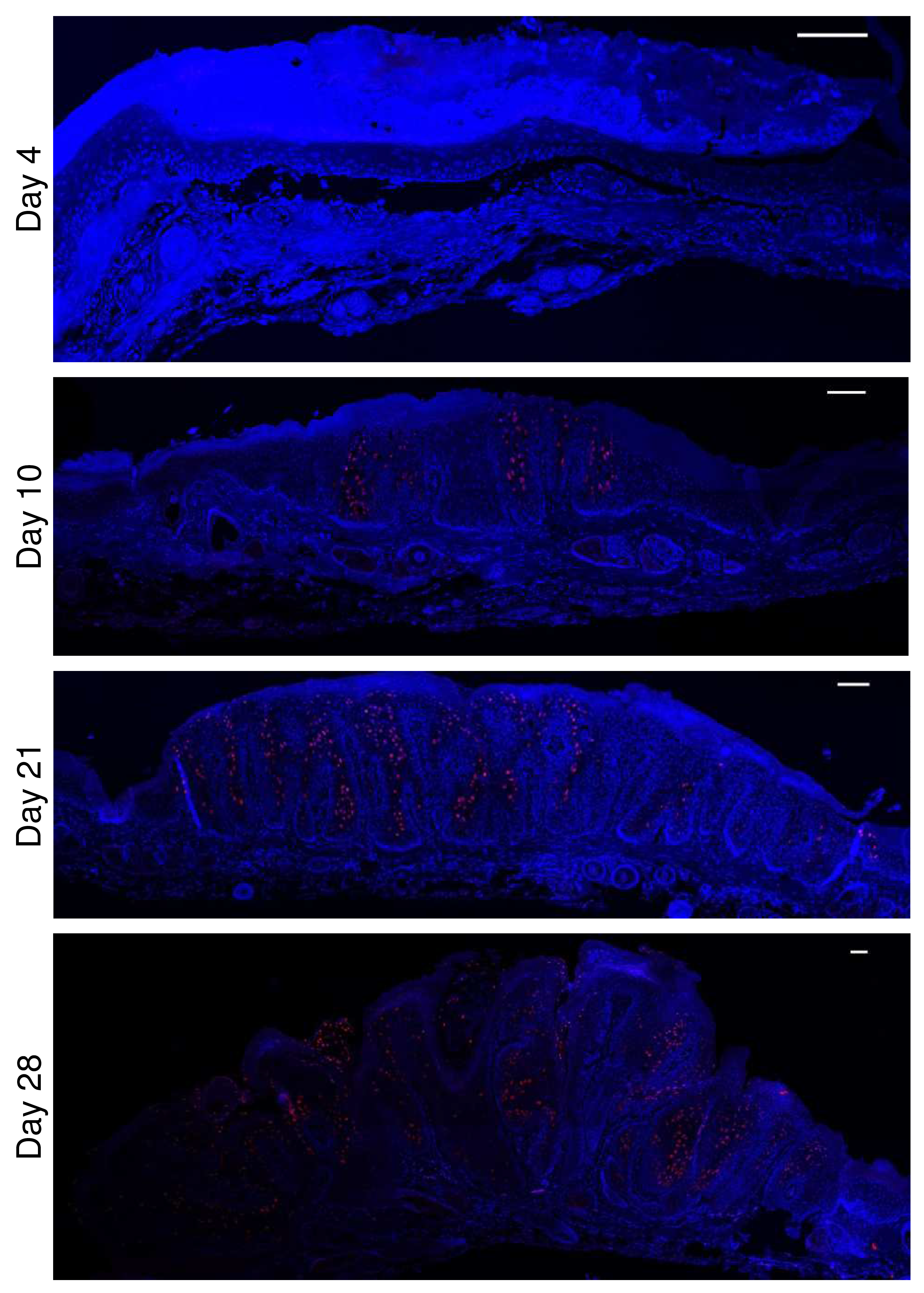

Supplement: S7 Fig — Nuclei (blue) were counterstained with Hoechst. See other details in Fig 8A. Scale bar = 100 μm. (TIF) [file ppat.1006715.s007.tif]

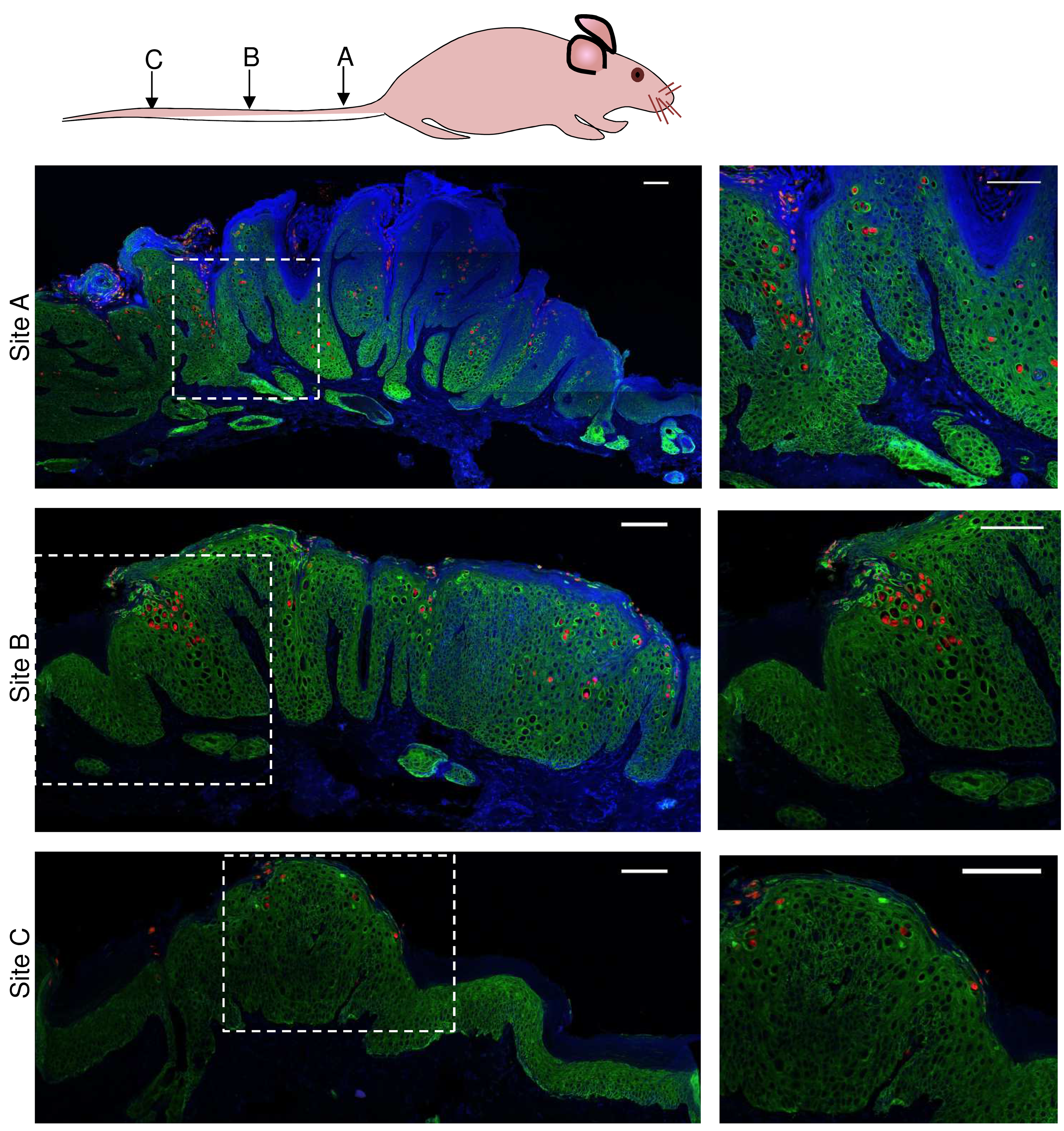

Supplement: S8 Fig — Each panel consists of a high-resolution wide-field image representing immunofluorescence detection of protein L1 (red) and keratins (Krt10 & Krt14, green) in the infected site accompanied by an inset showing a high magnification on the right. Scale bar = 100 μm. All sites at 28 days post-infection show clear evidence of papillomatosis accompanied with L1 protein expression. However, papillomas grew at different rates. (TIF) [file ppat.1006715.s008.tif]

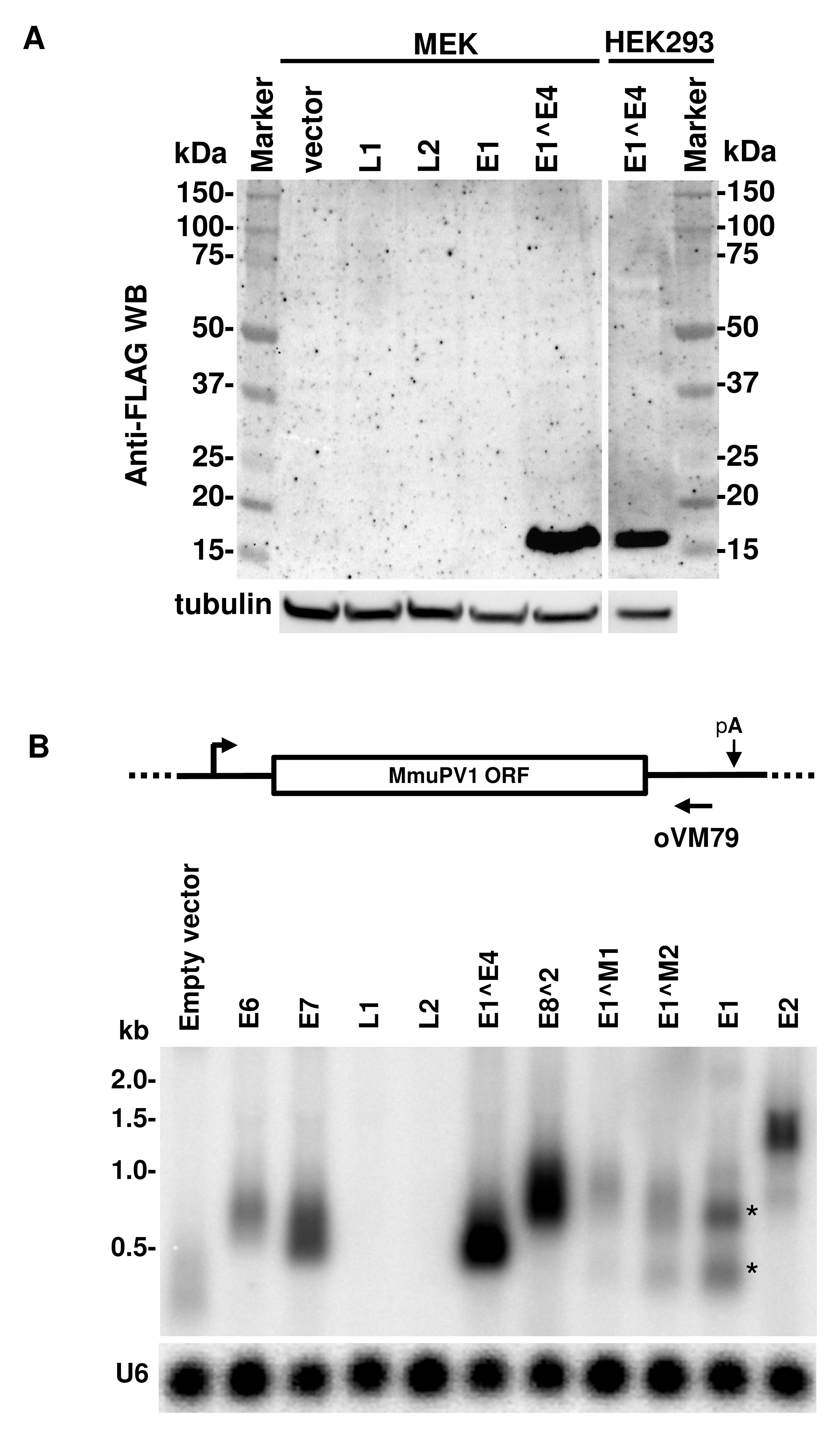

Supplement: S9 Fig — (A) Expression of MmuPV1 E1, E1^E4, L1 and L2 protein in mouse epithelial keratinocytes (MEK). MEK at 1 x 10^5^ in a 6-well plate were transfected with 1 μg of individual vectors expressing the indicated MmuPV1 protein as a Flag-fusion or with an empty vector pFLAG-CMV-5.1 (Sigma-Aldrich) as a negative control. Cell lysates prepared at 45 h after transfection were blotted with an anti-Flag antibody. Tubulin served as a loading control. (B) Expression level of individual MmuPV1 ORF RNAs in HEK293 cells. Total RNA (~5 μg) prepared 24 h after transfection of HEK293 (5 x 10^5^ cells per well) in a 6-well plate with 2 ug of individual plasmid DNA were examined by Northern blot with a 32P-labeled oligo probe (oVM79) hybridizing to a common 3’ UTR region as diagramed on the panel top. Empty vector was used as a negative control and U6 served as a sample RNA loading control. See Fig 9A for size estimation of individual ORF mRNAs. *, spliced E1 RNA. (TIF) [file ppat.1006715.s009.tif]
